# Nicotinamide riboside Induced Energy Stress and Metabolic Reprogramming in BEAS-2B Cells

**DOI:** 10.1021/acs.chemrestox.3c00312

**Published:** 2024-07-11

**Authors:** Everson Willian Fialho Cordeiro, Elisabete Leide Marzola, Ricardo Soei Maekawa, Matheus Relvas dos Santos, Lucas Gade Assunção, Mariana Pereira Massafera, Joseana de Oliveira, Thainá Gomes Cury Batista, Maria Cármen Oliveira Pinho de Sales, Silvya Stuchi Maria-Engler, Paolo Di Mascio, Marisa Helena Gennari de Medeiros, Graziella Eliza Ronsein, Ana Paula de Melo Loureiro

**Affiliations:** † Departamento de Análises Clínicas e Toxicológicas, Faculdade de Ciências Farmacêuticas, Universidade de São Paulo, Av. Prof. Lineu Prestes 580, CEP 05508-000 São Paulo, Brazil; ‡ Departamento de Bioquímica, Instituto de Química, Universidade de São Paulo, Av. Prof. Lineu Prestes 748, CEP 05508-000 São Paulo, Brazil

## Abstract

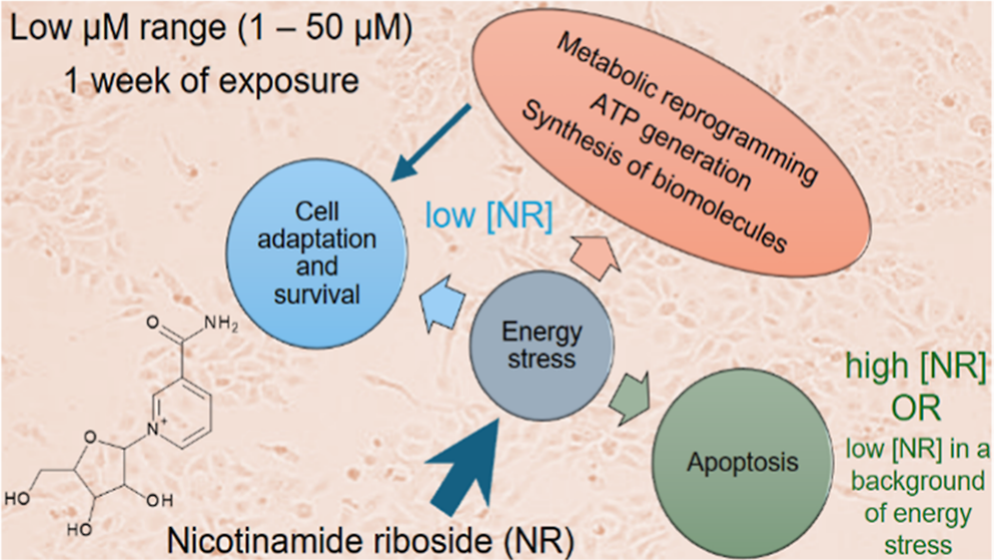

Nicotinamide riboside (NR), a NAD^+^ precursor, has received attention due to several health benefits it has induced in experimental models. Studies in cultured cells, animals, and humans consistently show increased NAD^+^ availability after NR supplementation, which is considered the only mode of NR action that leads to health benefits. In the present study, we show that a persistently low NR concentration (1 μM) in the growth medium of BEAS-2B human cells, grown in a monolayer, induces energy stress, which precedes a cellular NAD^+^ increase after 192 h. NR concentrations greater than 1 μM under the specified conditions were cytotoxic in the 2D cell culture model, while all concentrations tested in the 3D cell culture model (BEAS-2B cell spheroids exposed to 1, 5, 10, and 50 μM NR) induced apoptosis. Shotgun proteomics revealed that NR modulated the abundance of proteins, agreeing with the observed effects on cellular energy metabolism and cell growth or survival. Energy stress may activate pathways that lead to health benefits such as cancer prevention. Accordingly, the premalignant 1198 cell line was more sensitive to NR cytotoxicity than the phenotypically normal parent BEAS-2B cell line. The role of a mild energy stress induced by low concentrations of NR in its beneficial effects deserves further investigation. On the other hand, strategies to increase the bioavailability of NR require attention to toxic effects that may arise.

## Introduction

**Figure 1:**
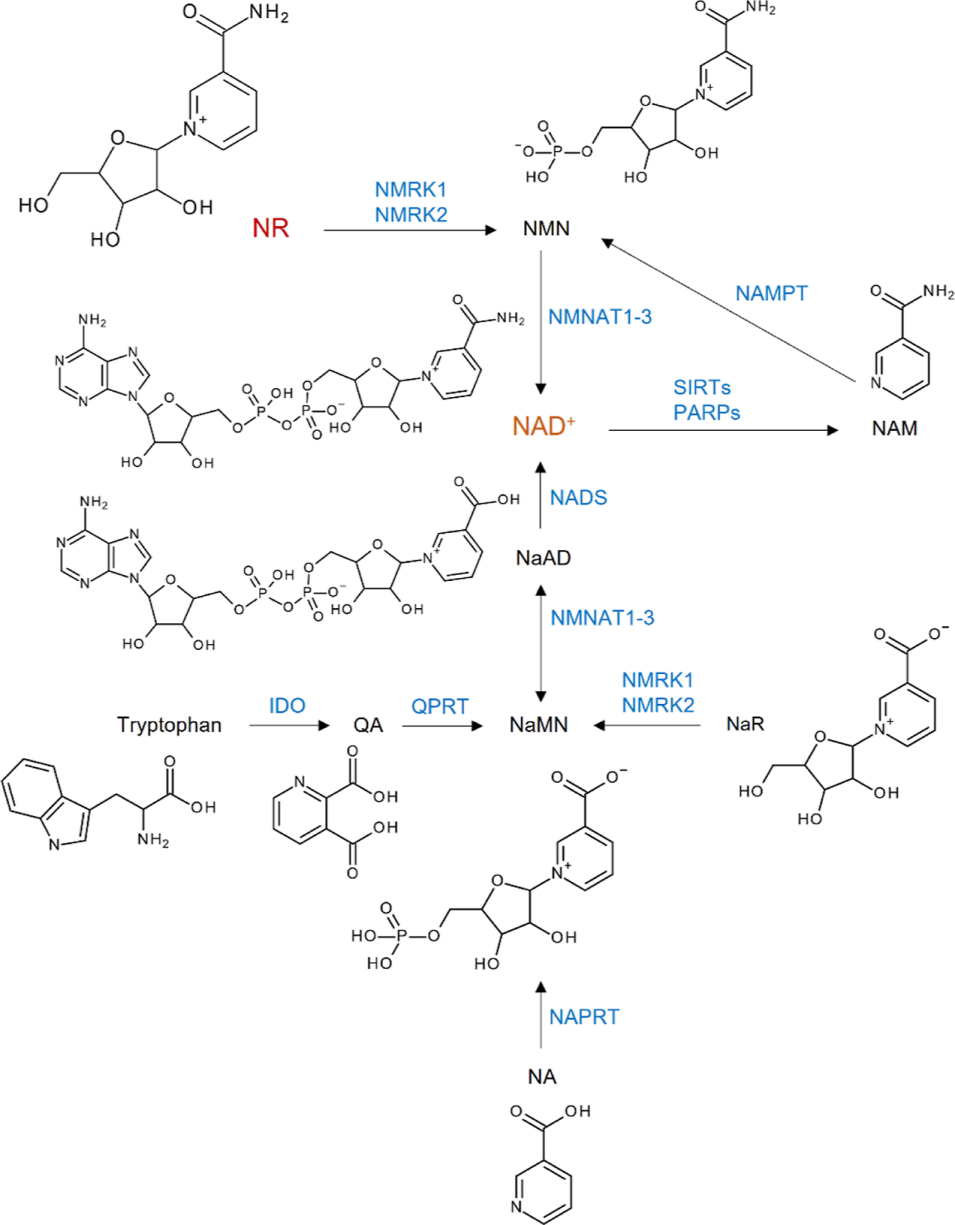
Pathways for NAD^+^ generation. QA, quinolinic acid; NaMN, NA mononucleotide; NA, nicotinic acid; NaR, NA riboside; NaAD, NA adenine dinucleotide; NAM, nicotinamide; NMN, NAM mononucleotide; NR, NAM riboside; NAD^+^, NAM adenine dinucleotide; IDO, indoleamine 2,3-dioxygenase; QPRT, quinolinic acid phosphoribosyl transferase; NAPRT, NA phosphoribosyltransferase; NMRK, NAM riboside kinase; NMNAT, NAM mononucleotide adenylyltransferase; NADS, NAD synthase; NAMPT, NAM phosphoribosyltransferase; SIRTs, sirtuins; PARPs, poly(ADP-ribose)polymerases.

Nicotinamide (NAM) riboside (NR, Figure 1) is a substrate for NAM adenine dinucleotide (NAD^+^) biosynthesis in mammalian, fungal, and bacterial cells.^1^ It is found in small amounts (∼5 μM) in milk^2,3^ and is now considered a NAD^+^ precursor vitamin in vertebrates, as are nicotinic acid (NA) and NAM, which are collectively known as niacin or vitamin B3.^4^ Other NAD^+^ precursors are NAM mononucleotide (NMN), NA mononucleotide (NaMN), NA riboside (NaR), NA adenine dinucleotide (NaAD), and tryptophan.^5,6^Figure 1 summarizes the pathways for NAD^+^ generation from different precursors.^7^

Among NAD^+^ precursors, NR and NAM mononucleotide are considered promising for clinical use. These molecules are water-soluble and orally bioavailable. However, it is possible that NAM mononucleotide is not transported across the cell plasma membrane and that its effects are due to its extracellular degradation into membrane-permeable precursors, such as NR.^5,6^ On the other hand, experimental studies have shown that NR is quickly converted into NAM in plasma, in culture medium supplemented with fetal bovine serum (FBS), and in the liver during first pass metabolism.^6^

NAD^+^ and NADH play important roles in regulating the activities of the glycolytic pathway, tricarboxylic acid cycle, and mitochondrial electron transport chain, ultimately regulating adenosine triphosphate (ATP) production.^8^ In addition to its essential function as a coenzyme in redox reactions, NAD^+^ is a cosubstrate for the activities of adenosine diphosphate (ADP)-ribosyltransferases, poly(ADP-ribose)polymerases, sirtuins (SIRTs), and cyclic ADP-ribose synthases, such as CD38 and CD157.^9^ Thus, NAD^+^ participates in posttranslational modifications of proteins (ADP-ribosylation and deacetylation of lysine residues) and is a precursor to molecular messengers, such as ADP-ribose, cyclic ADP-ribose, and *O*-acetyl-ADP-ribose, involved in the mobilization of intracellular Ca^2+^ and subsequent signaling.^10^ It is implicated in the epigenetic control of gene transcription via histone deacetylation by SIRT1 and SIRT2 as well as in the activation of transcription factors regulated by deacetylation catalyzed by SIRT1.^10^ The activities of important components of the electron transport chain, fatty acid degradation, and ammonia detoxification pathways are also regulated by the mitochondrial SIRTs SIRT3, SIRT4, and SIRT5.^10^

It was verified that the exposure of different cell lines, namely, Neuro 2a (neuroblastoma), human embryonic kidney (HEK293), and AB1 (mouse embryonic stem cells), to NR (50–600 μM for 48 h) increased by as much as 2-fold the intracellular NAD^+^ concentration. A 2.7-fold increase in NAD^+^ content was also observed when the cells were exposed to 1 mM NR for 24 h without affecting cell survival.^1^ Similar increases in NAD^+^ content were obtained for the C2C12 (murine myoblasts), Hepa1.6 (murine hepatoma), and human embryonic kidney (HEK293T) cell lines exposed to 500 μM and 1 mM NR for 24 h.^11^ In another study, no cytotoxicity was observed for the AML12 mouse hepatocyte cell line exposed to 10 mM NR for 24 h.^12^

Genotoxicity studies using the Ames test, in vitro chromosomal aberration assay, and in vivo micronucleus assay revealed that NR is not mutagenic or clastogenic.^13^ A subchronic toxicity study in male and female rats revealed a no observed adverse effect level (NOAEL) of 300 mg/kg/day. The safe upper intake level of NR for humans was estimated to be 3 mg/kg/day (180 mg/day for 60 kg body weight) through the application of a 100-fold safety factor to the NOAEL of the subchronic study in rats. Among the toxic effects observed in the rats that received the highest NR dose (3000 mg/kg/day) were liver damage, thyroid follicular cell hypertrophy, nephropathy, and hypertrophy of zona glomerulosa in adrenals.^13^

NR is available as a supplement (>99% NAM riboside chloride). Ninety-three clinical trials evaluating NR were found in the ClinicalTrials.gov database (accessed on March 12, 2024), with some adverse effects observed in a few studies that presented results. However, different studies indicate that the supplementation with NR is safe and increases the NAD^+^ levels and metabolism in circulating peripheral blood mononuclear cells (PBMCs).^14−17^

Reported benefits of NR supplementation in experimental models include protection against noise-induced hearing loss,^18^ improvement of cognitive function in Alzheimer’s disease,^19^ treatment of mitochondrial myopathy,^20^ reduction in blood glucose and hepatic steatosis in diabetic mice,^21^ protection against diabetic neuropathy,^21^ amelioration of hepatic low-grade chronic inflammation,^22^ protection against high-fat-diet-induced metabolic abnormalities,^11^ prevention of lung and heart injury in sepsis,^23^ treatment of age-related ovarian infertility,^24^ protection against retinal degeneration,^25^ and inhibition of hepatocellular carcinoma growth.^26^

In vitro cell culture models can assist in understanding the mechanisms by which NR induces the observed beneficial effects. They can also assist in the assessment of the concentration required in a cell compartment to attain the desired effects. To date, few in vitro investigations have been performed after 24 or 48 h of a single exposure of the cell culture to NR in the 10 μM to 17 mM concentration range,^1,11−13^ which are remarkably high concentrations compared to the concentrations found in whole human blood, even after NR supplementation. The whole blood NR concentration was 0.16 μM after 21 days of 1000 mg/day of NR supplementation in aged men, which did not differ from the blood concentration detected in the placebo treatment.^27^ In a pharmacokinetics study of NR, its mean basal level in the blood of healthy subjects was 0.023 μM. The mean average concentration at steady state 9 days after NR dosing (dose escalation from 250 to 2000 mg/day over 9 days) increased to 0.04 μM.^16^ The concentration of NR found in mouse serum was 0.007 μM.^28^

Here, we present data showing that daily exposure of the BEAS-2B human cell line (derived from normal human bronchial epithelium) to NR in the 1–50 μM concentration range over 192 h led to cytotoxicity when the concentrations exceeded 1 μM in the culture grown in a monolayer. We also show that BEAS-2B cell spheroids exposed to NR over 168 h presented increased apoptosis for all concentrations tested (1–50 μM). Taking into account that the bronchial epithelial cell growth medium contains 0.3 μM NAM as the NAD^+^ precursor,^29^ the addition of 1 μM NR represents a 4.3-fold increase in the availability of an NAD^+^ precursor to the cells. NR induced changes in cell energy metabolism over the incubation period in the 2D and 3D culture models and an evident increase in cellular NAD^+^ only after 192 h in the 2D model. Shotgun proteomics allowed us to observe that exposure to NR modulated the abundance of proteins that agree with the effects observed on cellular energy metabolism and cell growth or survival. We further compared the cytotoxicity of NR (0.5–2 μM over 168 h) induced in the BEAS-2B cell line and its premalignant derivative, the 1198 cell line. The 1198 cell line was obtained from tumors grown in the subcutaneous dorsal tissue of nude mice into which BEAS-2B cells exposed to cigarette smoke condensate had been transplanted.^30^ The premalignant 1198 cells were more sensitive than the BEAS-2B cells to the cytotoxic effect of NR. These data can aid in the research of a safe and effective dose of NR for human supplementation.

## Results

### NR Stability

**Figure 2:**
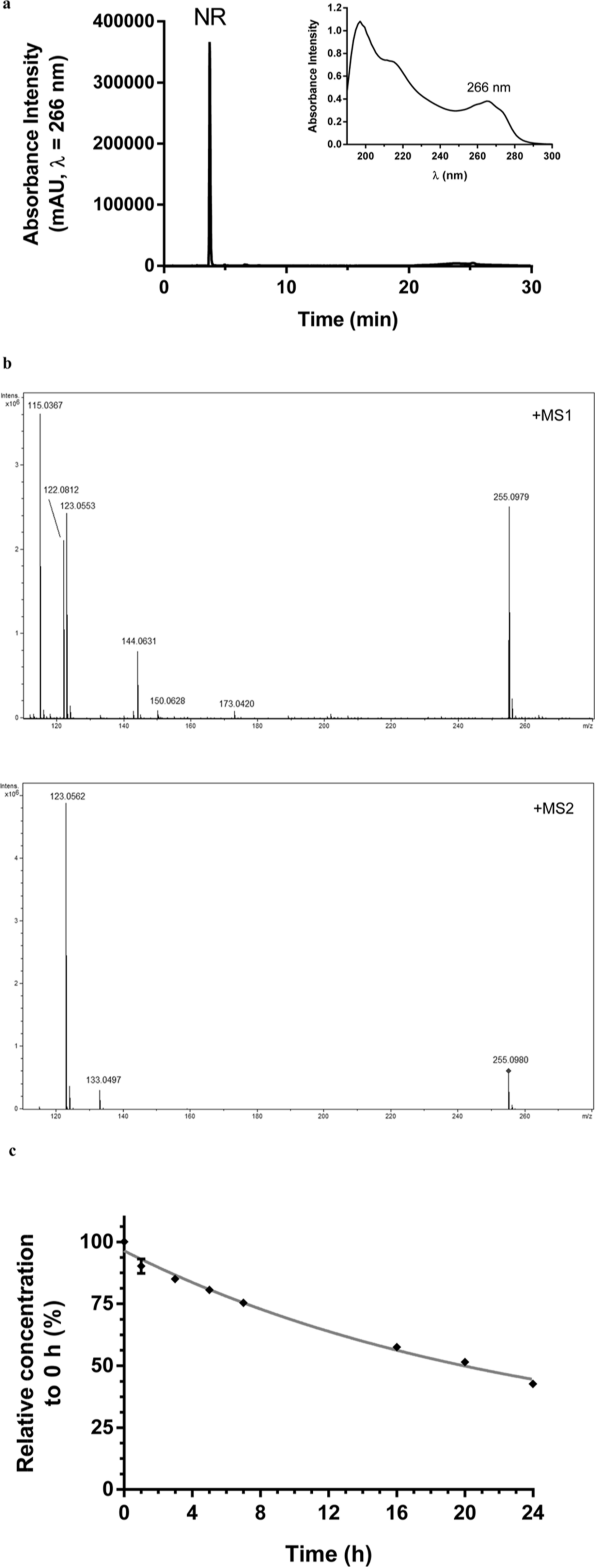
NR obtained from alkaline phosphatase hydrolysis of the NAM mononucleotide. (a) Chromatogram of the purified NR (λ = 266 nm) and its absorbance spectrum. (b) NR high-resolution mass spectra obtained in MS1 and MS2. (c) NR stability assessment in the cell-free culture medium at 37 °C.

NR obtained via alkaline phosphatase hydrolysis of NAM mononucleotide was purified by high performance liquid chromatography coupled to photodiode array detector (HPLC-PDA) (Figure 2a). The solution containing the purified molecule was directly injected into a high-resolution mass spectrometer (maXis 3G QTOF, Bruker Daltonics, Bremen, Germany) for structural characterization (Figure 2b and Table 1). Aliquots of the aqueous stock solution were maintained at −80 °C until use. The NR concentration in the frozen stock solution was monitored for 90 days by assessing the absorbance at 266 nm. The mean ± SD absorbance intensity was 0.43 ± 0.01, with a coefficient of variation of 3.4%. Absorbance intensity at day 1 was 0.422 and at day 90 was 0.419. NR was stable under the stock conditions.

**Table 1:**
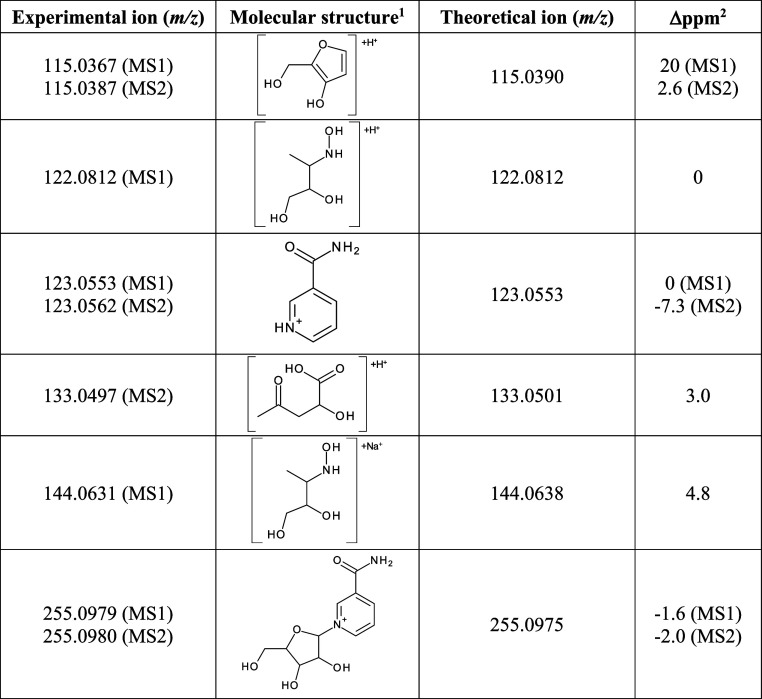
Ions Detected in the NR High-Resolution Mass Spectra Obtained in Positive Mode and the Corresponding Molecular Structures

1 METLIN was used for molecular structure search.

2 Δppm, mass error = [(theoretical *m*/*z* – experimental *m*/*z*)/theoretical *m*/*z*] × 10^6^.

NR stability in the cell-free culture medium over 24 h at 37 °C was also monitored. The data obtained via HPLC-PDA revealed that NR was 24% degraded within 7 h, 42% within 16 h, and 57% within 24 h, presenting an estimated half-life (*t*_1/2_) of 20 h (Figure 2c). The culture medium containing NR was then renewed every 24 h in the cell culture experiments to ensure the bioavailability of NR throughout the incubation period.

### NR Cytotoxicity to BEAS-2B Cells Grown in a Monolayer

**Figure 3:**
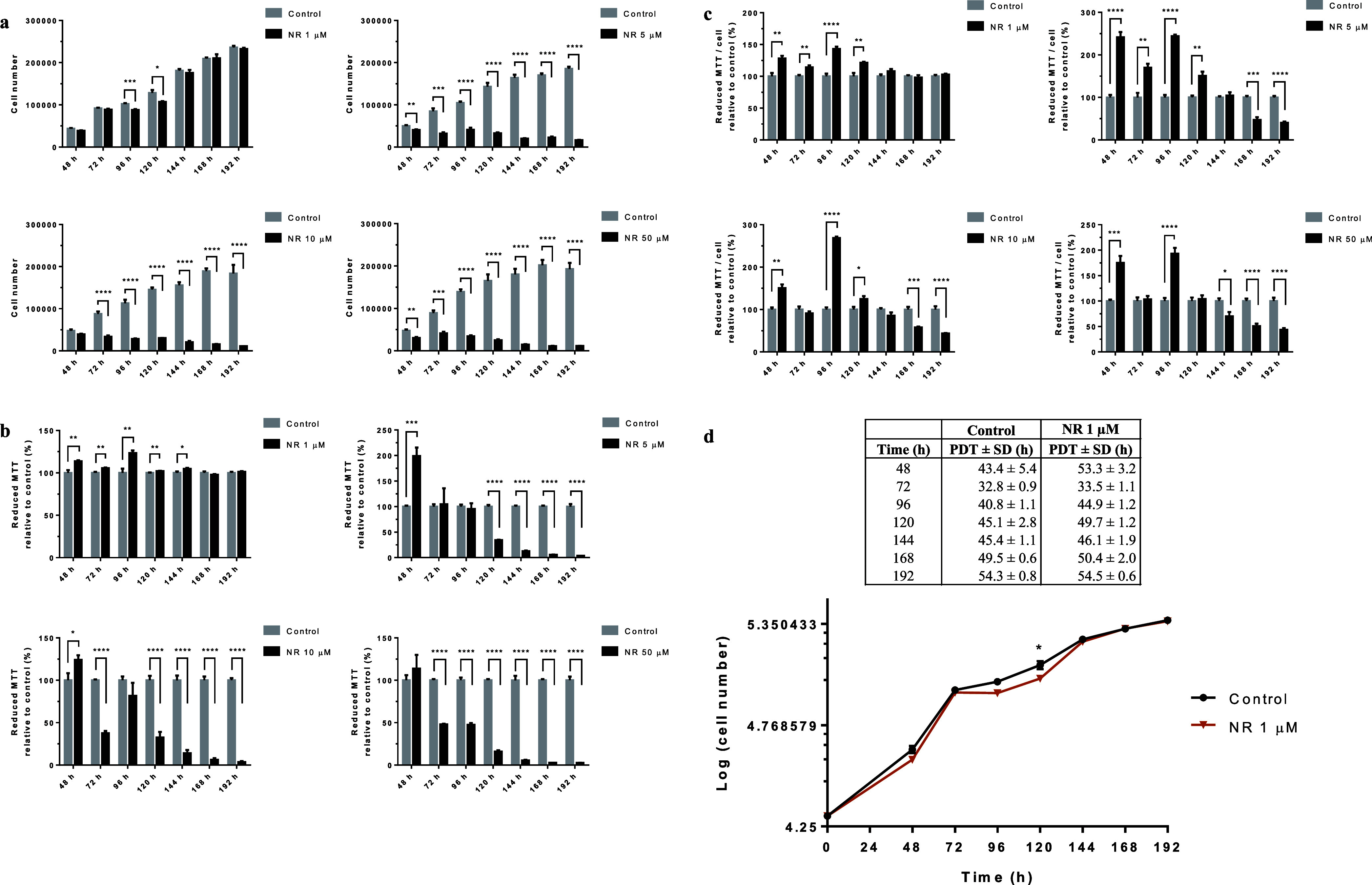
NR affects BEAS-2B cell growth at concentrations above 1 μM in the culture grown in a monolayer. (a) CVD assay of the cells exposed to the indicated NR concentrations in the time range from 48 to 192 h. (b) Cell viability assessed by the MTT assay. (c) Reductive capacity of MTT in each cell. (d) Growth curves of the cells exposed or not to 1 μM NR. PDT, cell population doubling time. Statistics of (a–c): unpaired *t*-test, *N* = 4 or 5. The experiments with the different NR concentrations were performed independently in different periods. Statistics of (d): multiple *t* tests. *p < 0.05, **p < 0.01, ***p < 0.001, ****p < 0.0001.

NR cytotoxicity induced in BEAS-2B cells grown in a monolayer (2D cell culture model) was assessed every 24 h from the 48th to the 192nd hour. NR was tested in the concentration range from 1 to 50 μM using the crystal violet dye (CVD) and MTT assays. As shown in Figure 3a (the CVD assay results), NR concentrations of 5, 10, and 50 μM stopped cell growth and induced cell death from the 48 or 72 h incubation periods. Although some cell growth arrest was noticed after 96 and 120 h of exposure to 1 μM NR, the cells grew normally during the other periods of exposure to this concentration, which was considered noncytotoxic.

**Figure 4:**
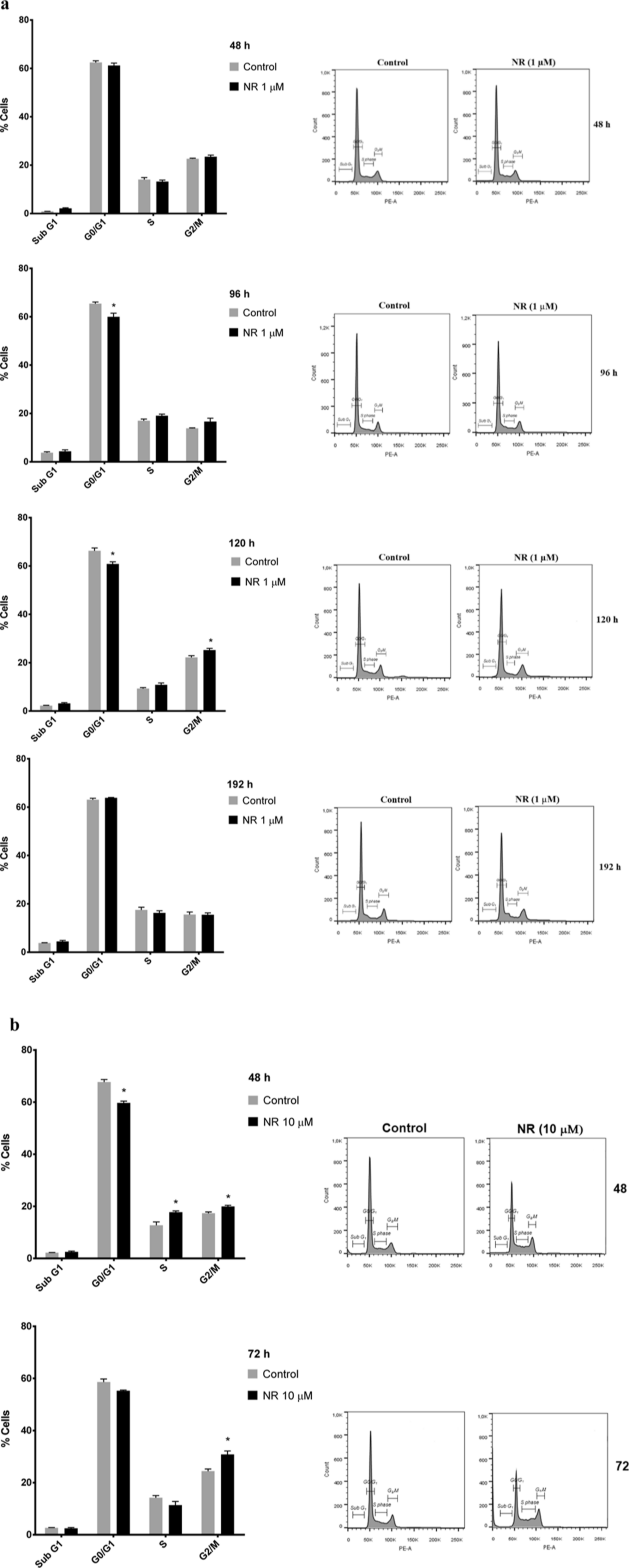
NR arrests BEAS-2B cell cycle in the G2/M phase depending on the concentration and the exposure duration in the culture grown in monolayer. Effects of (a) 1 μM NR and (b) 10 μM NR on the cell cycle at the indicated time points. Only cells adhered to the culture plates were used. Asterisks indicate the significant differences between the exposed and control cells in each cell cycle phase. Multiple *t* tests in each time point, *N* = 4, *p < 0.05.

The cell cycle was monitored at 48, 96, 120, and 192 h of exposure to 1 μM NR and at 48 and 72 h of exposure to 10 μM NR. Cell cycle arrest in G_2_/M was observed at 120 h of exposure to 1 μM NR (Figure 4a) and at 48 and 72 h of exposure to 10 μM NR (Figure 4b), which agreed with the growth arrest data obtained at the same time points and shown in Figure 3a.

As this observation was not in agreement with the literature on the cytotoxicity of NR, we performed the MTT assay as previously used for this assessment.^1^ The data obtained by the MTT assay are shown in Figure 3b. In agreement with previously published data, all the concentrations tested were considered noncytotoxic for the first 48 h of exposure. However, cytotoxicity was observed after 120 h of exposure to 5 μM NR and after 72 h of exposure to 10 or 50 μM NR, with renewal of the culture medium containing NR every 24 h. The MTT assay reflects the reduction of MTT dye to insoluble formazan crystals, which can occur via cellular oxidoreductases that use NADH or reduced nicotinamide adenine dinucleotide phosphate (NADPH).^31^ Therefore, the assay data may not reflect cytotoxicity if a parallel increase in oxidoreductase activities occurs during the assessment. This oxidoreductase activity is to be expected after cell exposure to an NAD^+^ precursor such as NR.

Considering the number of living cells at each time point and the corresponding MTT assay data, it was possible to calculate the reductive capacity of MTT in each cell. As shown in Figure 3c, NR increased the cellular MTT reductive capacity in the period from 48 to 120 h, and the capacity returned to normal or decreased in subsequent incubation periods, depending on the NR concentration tested.

The growth curves of the cells exposed to the noncytotoxic NR concentration of 1 μM and of the control cells are shown in Figure 3d together with the cell population doubling time calculated for each time point, starting with the initial plating of 2 × 10^4^ cells.

We further checked the cytotoxicity of NR using the commercially available NR chloride from Sigma-Aldrich (Cat. number SMB00907, CAS number 23111-00-4). The BEAS-2B cells were exposed daily to 10, 50, and 100 μM of the commercial molecule, and the CVD assay was performed at 24, 48, 72, 96, and 120 h of exposure. The crystal violet-stained cells were photographed prior to the final step of the assay. As shown in Figure S1, NR chloride stopped cell growth from 72 h and changed the cell morphology.

### NR Effects on the NAD^+^ Content, Energy Metabolism, and Protein Abundance in BEAS-2B Cells Grown in a Monolayer

The intracellular NAD^+^, ATP, ADP, and AMP contents were assessed after 25, 27, 72, 96, 120, 144, 168, and 192 h of BEAS-2B cell exposure to 1 μM NR, with renewal of the culture medium containing NR every 24 h.

**Figure 5:**
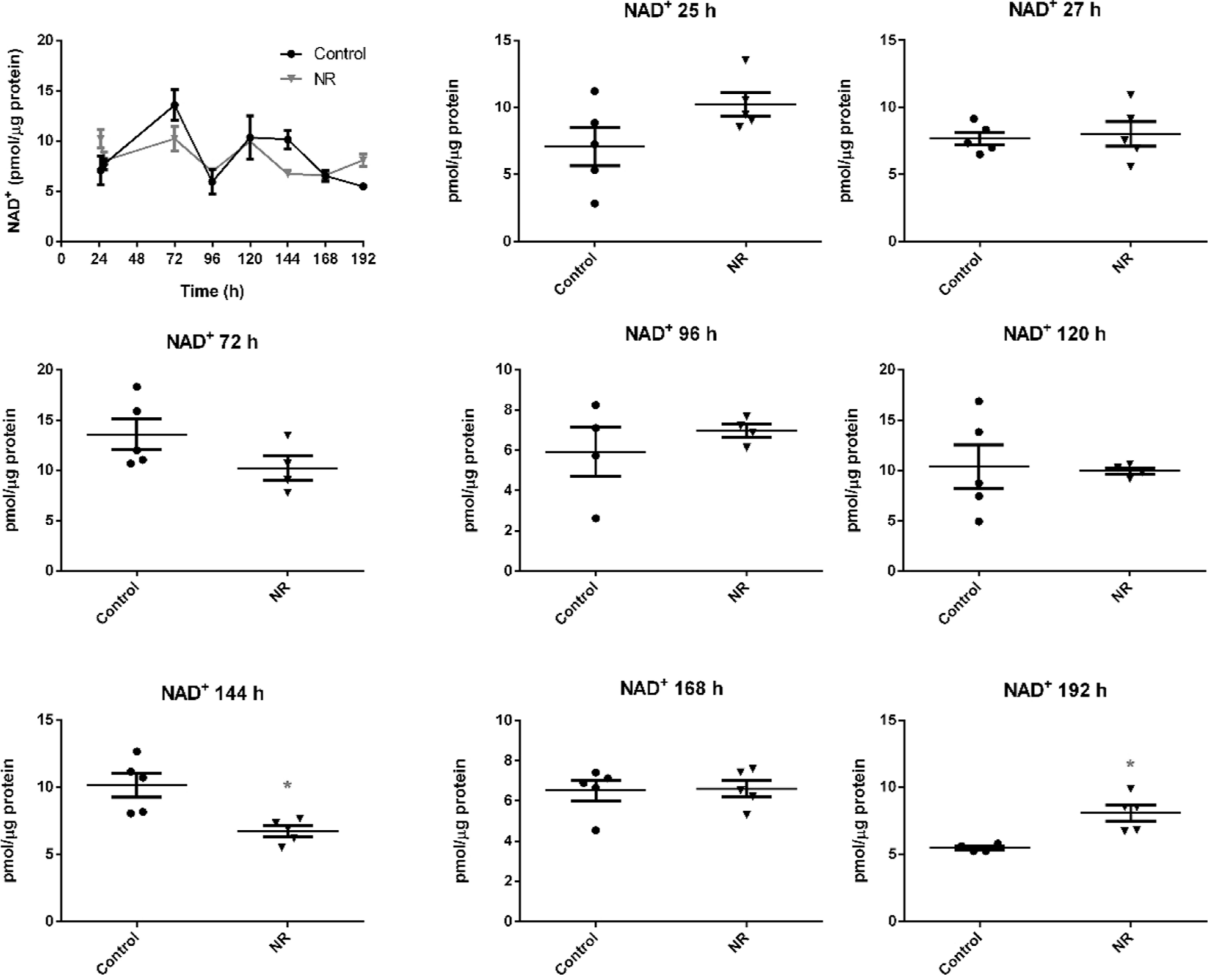
NR at the daily concentration of 1 μM increases BEAS-2B cells NAD^+^ content only after 192 h in the culture grown in a monolayer. The first panel gives the general view in the 24–192 h range. The other panels show the comparisons between the control and the NR exposed cells in each time point. Unpaired *t*-test with Welch’s correction, *N* = 4 or 5. *p < 0.05. The experiment results are representative of two independent experiments performed in the interval of 6 months.

**Figure 6:**
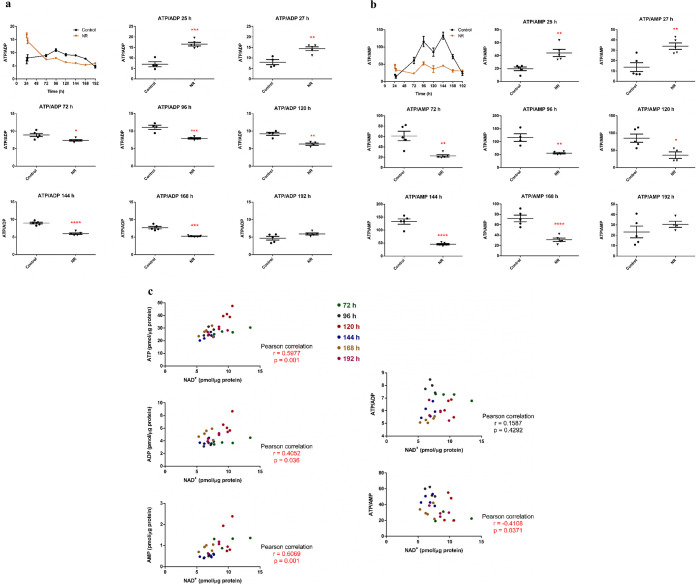
NR at the daily concentration of 1 μM induces energy stress in BEAS-2B cells from 72 to 168 h of exposure in the culture grown in a monolayer. The first panel in (a,b) gives the general view in the 24–192 h range. The other panels show the comparisons between the control and the NR exposed cells in each time point. (a) ATP/ADP ratios. Unpaired *t*-test with Welch’s correction, *N* = 4 or 5. (b) ATP/AMP ratios. Unpaired *t*-test with Welch’s correction, *N* = 4 or 5. (c) Correlation analyses of the levels of ATP, ADP, AMP, ATP/ADP, and ATP/AMP with the NAD^+^ content in the NR-exposed cells in the 72–192 h interval. The significant correlations are shown in red. *p < 0.05, **p < 0.01, ***p < 0.001, ****p < 0.0001. The experiment results are representative of two independent experiments performed in the interval of 6 months.

The increase in intracellular NAD^+^ was evident at 192 h, while a decrease was noticed at 144 h of exposure (Figure 5). In parallel, the ATP/ADP and ATP/AMP ratios of the exposed cells decreased in the interval from 72 to 168 h, with subsequent recovery at 192 h (Figure 6a,b). The cellular ATP, ADP, and AMP concentrations are shown in Table S1. The data show that a low daily noncytotoxic concentration of NR, which did not induce persistently detectable changes in cellular NAD^+^ levels, was able to modulate the energy metabolism of these cells. As pointed out in Figures 3a and 4a, cell growth arrest and cell cycle arrest were observed at 96 and 120 h of exposure to 1 μM NR, which may result from the energy stress suffered by the cells.

We also evaluated whether the levels of AMP, ADP, ATP, ATP/ADP, and ATP/AMP correlated with the NAD^+^ content in the control and NR-exposed cells in the 72–192 h interval. No correlation was observed in the control cells (Figure S2). However, the ATP, ADP, and AMP levels were positively correlated, and the ATP/AMP ratios were negatively correlated with the NAD^+^ content in the NR-exposed cells (Figure 6c). It is known that ATP is needed for NAD^+^ synthesis: 2 molecules of ATP are used for NAD^+^ synthesis from NR, and 4 molecules of ATP are used for NAD^+^ synthesis from NAM.^32^

**Figure 7:**
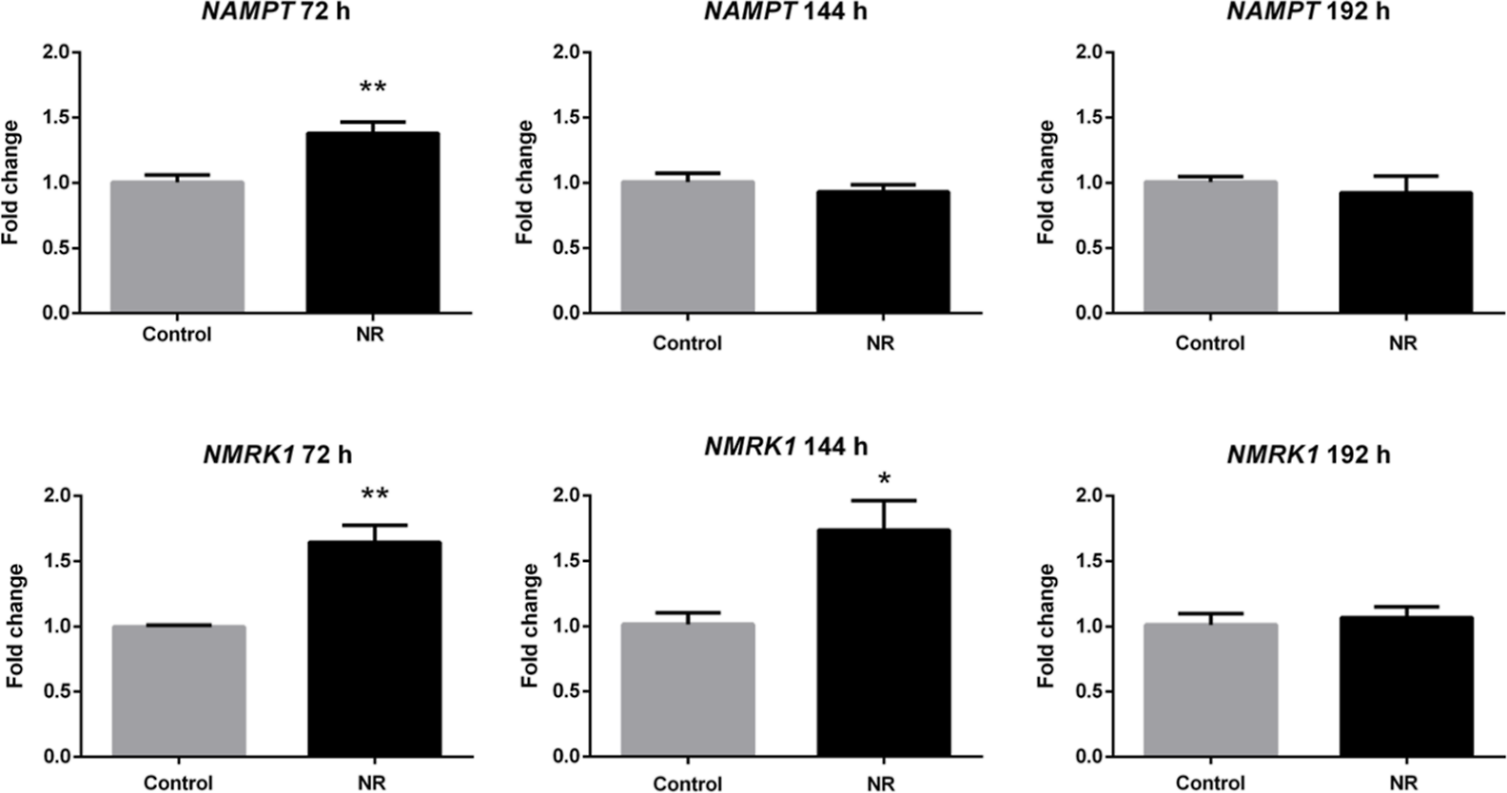
NR at the daily concentration of 1 μM induces the expression of NAM phosphoribosyltransferase (*NAMPT*) and NAM riboside kinase 1 (*NMRK1*) genes in BEAS-2B cells grown in a monolayer. Unpaired *t*-test, *N* = 5, *p < 0.05, **p < 0.01.

To verify if the cell ability to synthesize NAD^+^ from NR and NAM changed due to NR exposure, the expression levels of the NAM riboside kinase 1 (*NMRK1*) and NAM phosphoribosyltransferase (*NAMPT*) genes were quantified. The cDNA levels were quantified at 72, 144, and 192 h of exposure to 1 μM NR with the culture medium containing NR renewed every 24 h (Figure 7). The expression of both genes was induced in the presence of NR at 72 h, but only *NMRK1* expression was persistently induced at 144 h. The expression of *NMRK1* and *NAMPT* returned to the control levels at 192 h.

**Figure 8:**
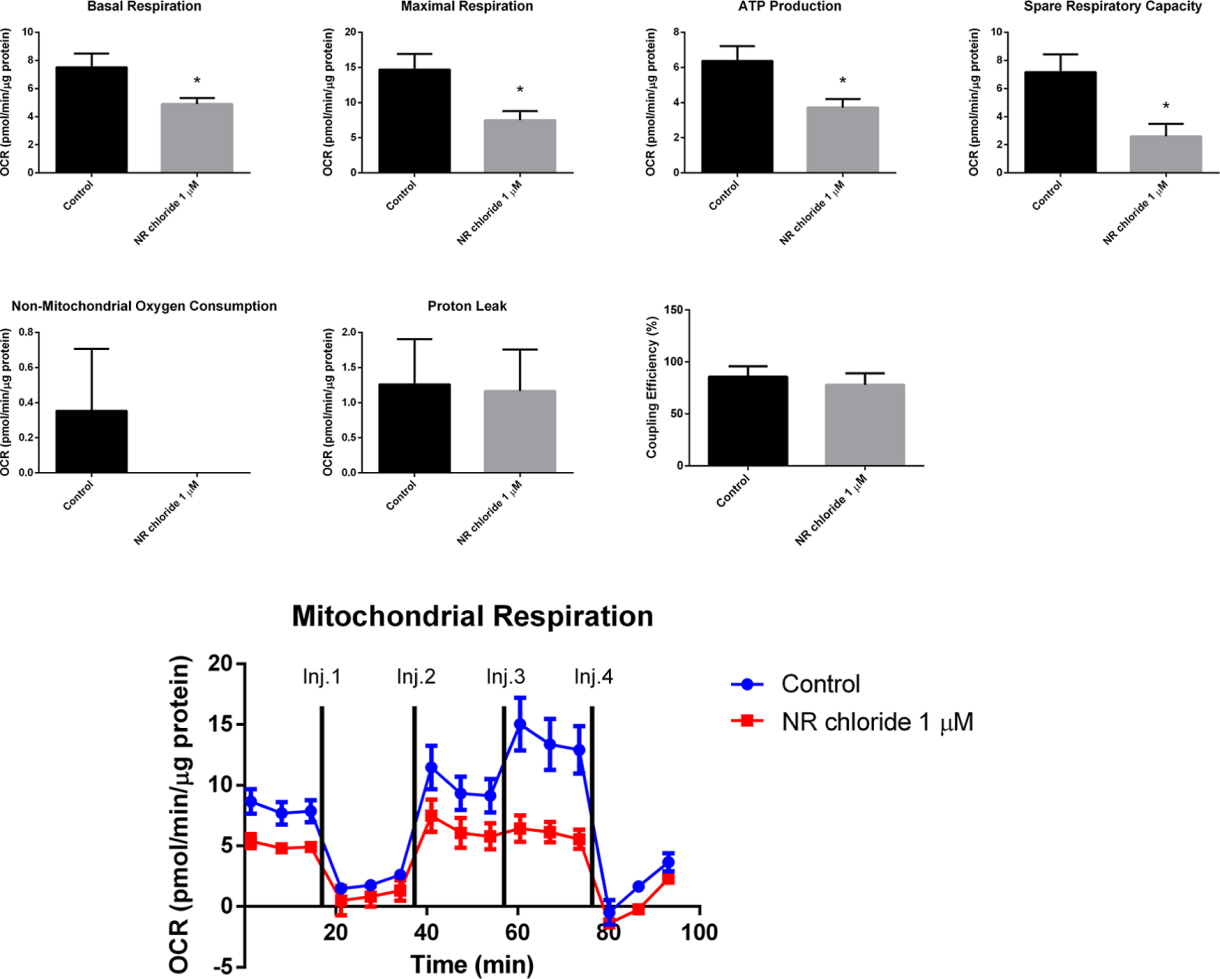
NR chloride at the daily concentration of 1 μM decreases mitochondrial respiration in BEAS-2B cells after 96 h of exposure in the culture grown in a monolayer. Oxygen consumption rate was detected under basal conditions followed by the sequential addition of oligomycin (Inj. 1), CCCP (two injections, Inj. 2 and Inj. 3), and rotenone + antimycin A (Inj. 4). Basal respiration, maximal respiration, ATP production rate, spare respiratory capacity, nonmitochondrial oxygen consumption, proton leak, and coupling efficiency were calculated using the Wave Desktop and Controller 2.6 software of the Seahorse analyzer. Unpaired *t*-test; control, *N* = 3; NR chloride, *N* = 4. *p < 0.05.

The effect of 1 μM NR chloride (Sigma-Aldrich, Cat. number SMB00907, CAS number 23111-00-4) on BEAS-2B cell mitochondrial respiration was analyzed using the mitochondrial stress test assay of the Seahorse Analyzer. After 96 h of exposure to 1 μM NR chloride, the BEAS-2B cells presented decreased basal respiration, decreased maximal respiration, decreased spare respiratory capacity, decreased ATP production rate, unchanged proton leak, unchanged nonmitochondrial oxygen consumption, and unchanged coupling efficiency (Figure 8). So, NR induced energy stress by decreasing the BEAS-2B cell mitochondrial respiration, which was not accompanied by an increase of nonmitochondrial respiration at 96 h of exposure.

We investigated the possible occurrence of reductive stress in NR supplementation by quantifying intracellular lactate and the generation of malondialdehyde as a marker of lipid peroxidation. As seen in Figure S3, daily exposure of the BEAS-2B cells to 1 μM NR for 192 h did not increase the concentrations of lactate and protected against malondialdehyde generation along the exposure period. This suggests that cellular exposure to NR under the defined conditions did not lead to excessive NADH generation.

**Figure 9:**
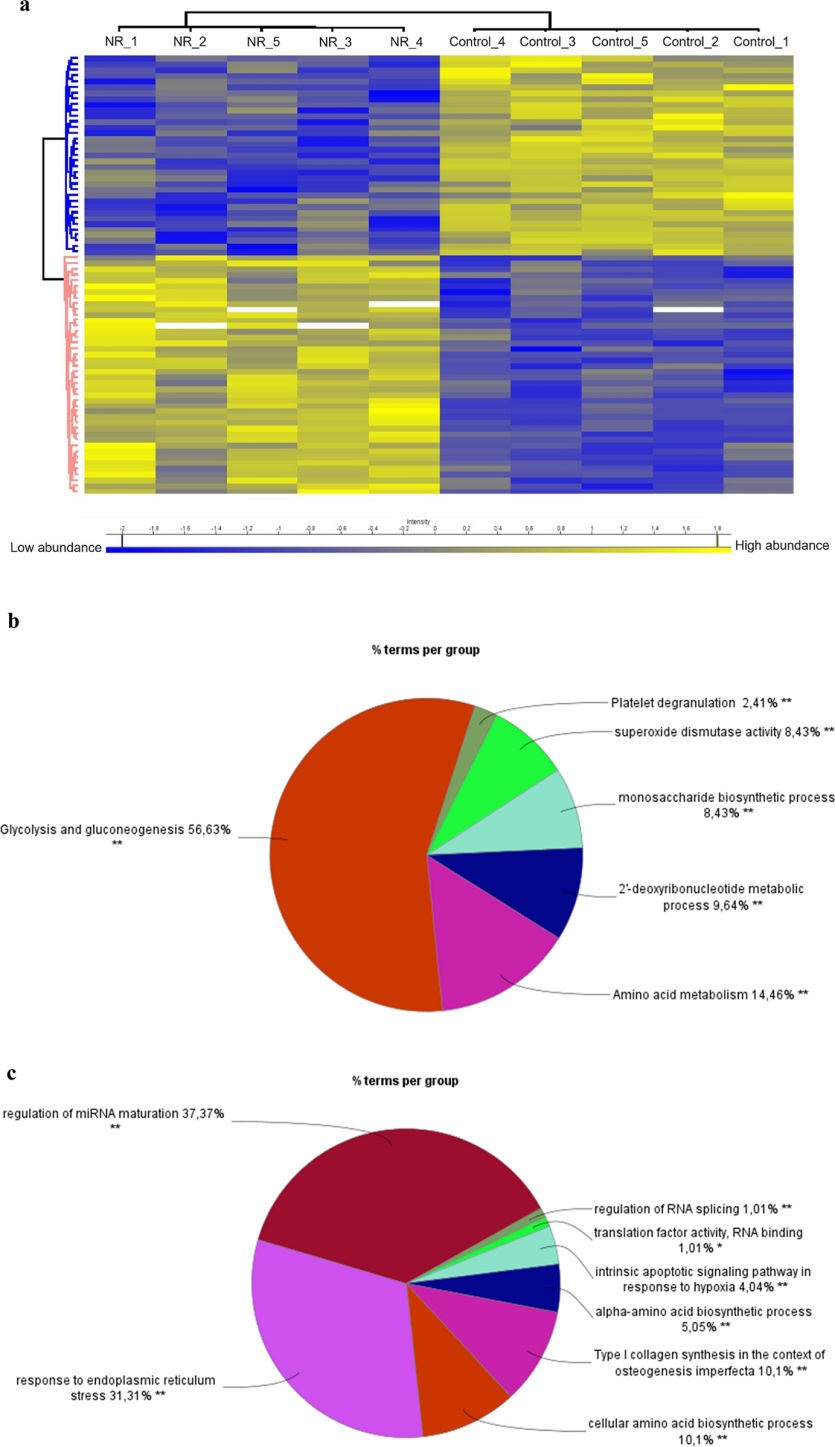
NR at the daily concentration of 1 μM modulates the abundance of proteins in BEAS-2B cells after 144 h of exposure in the culture grown in a monolayer. (a) Heatmap showing the 77 differentially abundant proteins (42 proteins more abundant in the NR group and 35 proteins more abundant in the control group). The protein names and gene symbols for each row of the heatmap are displayed in the same order in Table S2. Yellow, high abundance; blue, low abundance. (b) Overview of the significant groups of biological processes and pathways that resulted from the pathway enrichment analysis of the 42 more abundant proteins in the NR group. (c) Overview of the significant groups of biological processes and pathways that resulted from the pathway enrichment analysis of the 35 less abundant proteins in the NR group. Perseus software (version 2.0.11) was used for proteomics data processing and heatmap visualization. The ClueGo tool (version 2.5.10) of the Cytoscape software (version 3.10.1) was used for pathway enrichment analyses. Ontologies selected: WikiPathways, REACTOME_Pathways, GO_BiologicalProcess.

We further analyzed the protein abundance by shotgun proteomics of the BEAS-2B cells exposed daily to 1 μM NR for 144 h, compared to the control group. Among 2891 proteins identified in all samples, 1911 proteins were detected in at least three samples of each group (control and NR). After comparison between groups with an unpaired *t*-test and adjustment of the p value for multiple comparisons with a false discovery rate (FDR) of 0.05, we found 77 differentially abundant proteins. Among them, 42 were more abundant in the NR group, and 35 were more abundant in the control group (Figure 9a and Table S2).

Pathway enrichment analysis revealed that NR exposure increased the abundance of proteins that were significantly enriched on biological processes or pathways grouped under the terms glycolysis and gluconeogenesis, amino acid metabolism, 2′-deoxyribonucleotide metabolic process, monosaccharide biosynthetic process, superoxide dismutase activity, and platelet degranulation (Figures 9b, S4, and Table S3). The majority of the terms was grouped as glycolysis and gluconeogenesis, including glycolysis and gluconeogenesis related terms, as well as the terms manipulation of host energy metabolism, clear cell renal cell carcinoma pathways, metabolic reprogramming in colon cancer, NAD metabolism in oncogene-induced senescence and mitochondrial dysfunction-associated senescence, glycolysis in senescence, generation of precursor metabolites and energy, NADH regeneration, nucleotide phosphorylation, nucleotide metabolic process-related terms, ATP generation from ADP, ATP metabolic process, and ADP metabolic process (Figure S4). It seems that the BEAS-2B cells responded to the decrease in mitochondrial respiration and the consequent energy stress induced by NR with an increase of glycolysis for ATP generation and biomolecule synthesis at 144 h of exposure.

**Figure 10:**
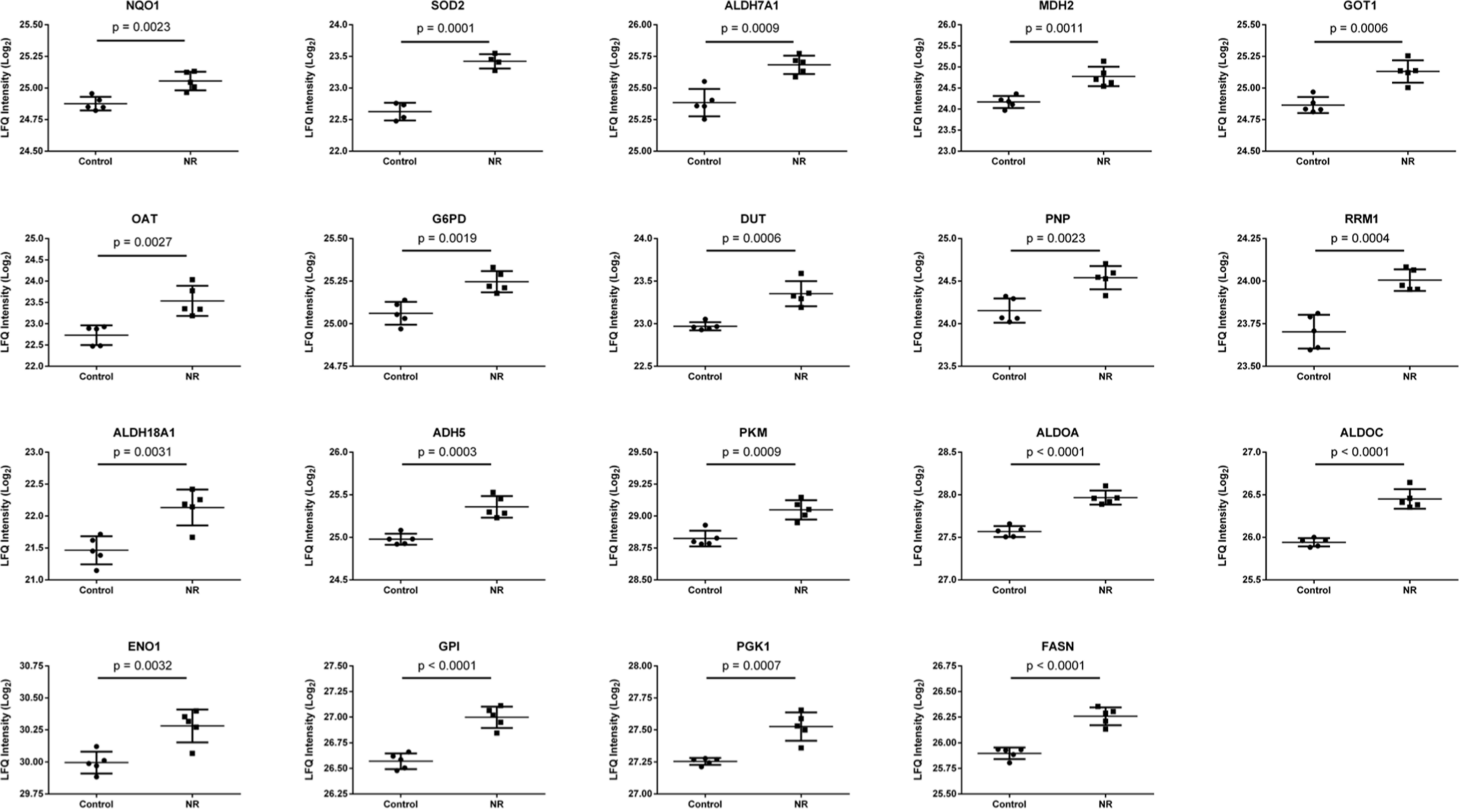
Nineteen proteins associated with 46 representative terms and pathways selected among the significant terms and pathways of Table S3 and Figure S4). Selection criteria: at least 2 genes from the loaded list associated with a term (number genes) and representing at least 5% of the total number of genes in the term (% associated genes). The proteins belong to the cluster of 42 more abundant proteins in the NR group. See Table S4 for protein names and functions.

The data shown in Figure S4 encompass 27 proteins (64.29% of the input list of 42 more abundant proteins in the NR group) associated with 73 representative terms and pathways after p value significance selection criteria. The initial selection criteria applied to the set of 42 proteins were a minimum of 2 genes from the loaded list associated with a term (# genes) and that these genes represented at least 2% of the total number of genes in the term (% genes/term). This general selection criteria allowed the association of 35 proteins (83.33% of the input list of 42 more abundant proteins in the NR group) to 150 representative terms and pathways. Among the significant terms and pathways of Figure S4, we selected those with at least 2 # genes and 5% genes/term for a description of the associated proteins. Nineteen proteins associated with 46 representative terms and pathways were then selected (Figure 10 and Table S4). Among these proteins, six (31.6%) are part of the glycolytic pathway [pyruvate kinase PKM (PKM), fructose-bisphosphate aldolase A (ALDOA), fructose-bisphosphate aldolase C (ALDOC), alpha-enolase (ENO1), glucose-6-phosphate isomerase (GPI), phosphoglycerate kinase 1 (PGK1)]. Seven proteins (36.8%) use NAD^+^, NADP^+^, NADH, or NADPH as coenzymes [NAD(P)H dehydrogenase [quinone] 1 (NQO1), alpha-aminoadipic semialdehyde dehydrogenase (ALDH7A1), mitochondrial malate dehydrogenase (MDH2), glucose-6-phosphate 1-dehydrogenase (G6PD), delta-1-pyrroline-5-carboxylate synthase (ALDH18A1), alcohol dehydrogenase class-3 (ADH5), fatty acid synthase (FASN)]. Three proteins (15.8%) are involved in purine metabolism and deoxyribonucleotide metabolic process [mitochondrial deoxyuridine 5′-triphosphate nucleotidohydrolase (DUT), purine-nucleoside phosphorylase (PNP), ribonucleoside-diphosphate reductase large subunit (RRM1)]. Two proteins (10.5%) are pyridoxal phosphate-dependent enzymes with aminotransferase activity [cytoplasmic aspartate aminotransferase (GOT1), mitochondrial ornithine aminotransferase (OAT)]. One protein (5.3%) is mitochondrial superoxide dismutase (SOD2) (Figure 10 and Table S4).

The increased abundance of NAD(P)H dehydrogenase [quinone] 1 (NQO1), mitochondrial superoxide dismutase [Mn] (SOD2), and ALDH7A1 (Figure 10 and Table S4) indicates an adaptation of the NR exposed cells to increased generation of reactive oxygen species and lipid peroxidation-derived aldehydes. This is in line with the observed protection afforded by NR against the generation of malondialdehyde (Figure S3).

The increased abundance of five of the ten enzymes of the glycolytic pathway, including the two ATP-generating glycolytic enzymes—PGK1 and PKM—indicates the importance of glycolysis for cellular recovery from NR-induced energy stress. Yet, the more abundant enzymes mitochondrial MDH2 and cytoplasmic GOT1 are part of the malate-aspartate shuttle, important for converting NADH generated in the cytosol into mitochondrial NADH. Another more abundant enzyme in the NR-exposed cells was ADH5, which catalyzes the oxidation of long-chain primary alcohols, long chain omega-hydroxy fatty acids (such as 20-HETE), and *S*-(hydroxymethyl) glutathione, and generates NADH in the cytosol. ALDH7A1 is also involved in fatty acid degradation and generation of NADH in the cytosol. Furthermore, the also more abundant G6PD connects glycolysis to the pentose phosphate pathway, which provides NADPH for antioxidant activity and fatty acid synthesis, as well as ribose and deoxyribose phosphate for nucleotide and nucleic acid synthesis. According to this, the abundance of the enzymes FASN, DUT, PNP, and RRM1 was increased after NR exposure. The increased abundance of PNP can also contribute to increased degradation of NR to NAM and ribose-1-phosphate, competing with NR phosphorylation to NMN by NAM riboside kinase 1 (NMRK1) in the pathway of NAD^+^ synthesis shown in Figure 1.

In addition, the increased abundance of ALDH7A1, ALDH18A1, GOT1, mitochondrial OAT, and ADH5 points to the modulation of amino acid metabolism in BEAS-2B cells exposed to NR (Figure 10 and Table S4).

The same pathway enrichment analysis criteria were applied to the cluster of less abundant proteins in the NR-exposed cells. It was verified that NR exposure decreased the abundance of proteins that were significantly enriched on biological processes or pathways grouped under the terms regulation of miRNA maturation, response to endoplasmic reticulum stress, cellular amino acid biosynthetic process, type I collagen synthesis in the context of osteogenesis imperfecta, alpha-amino acid biosynthetic process, intrinsic apoptotic signaling pathway in response to hypoxia, translation factor activity - RNA binding, and regulation of RNA splicing (Figures 9c, S5, and Table S5). Most of the terms were grouped as regulation of miRNA maturation and in response to endoplasmic reticulum stress (Figure 9c).

**Figure 11:**
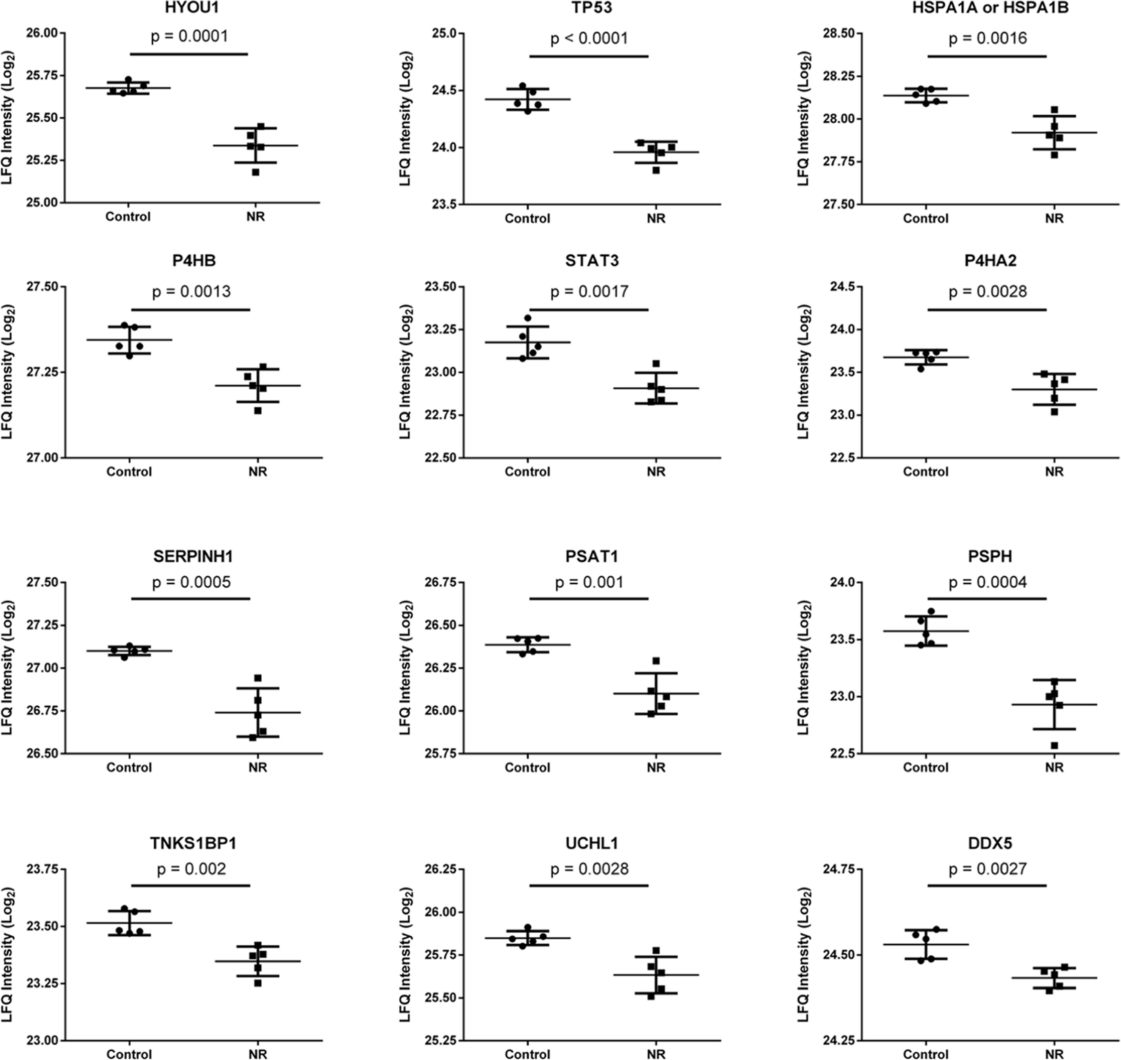
Twelve proteins associated with 36 representative terms and pathways selected among the significant terms and pathways of Table S5 and Figure S5. Selection criteria: at least 2 genes from the loaded list associated with a term (number genes) and representing at least 5% of the total number of genes in the term (% associated genes). The proteins belong to the cluster of 35 less abundant proteins in the NR group. See Table S6 for protein names and functions.

The data shown in Figure S5 encompass 23 proteins (65.71% of the input list of 35 less abundant proteins in the NR group) associated with 66 representative terms and pathways after p value significance selection criteria. The initial selection criteria (2 # genes and 2% genes/term) allowed the association of 25 proteins (71.43% of the input list of 35 less abundant proteins in the NR group) to 127 representative terms and pathways. Among the significant terms and pathways of Figure S5, we selected those with at least 2 # genes and 5% genes/term for a description of the associated proteins. Twelve proteins associated with 36 representative terms and pathways were then selected (Figure 11 and Table S6).

The 12 selected proteins have in common their role in the development of cancer. Except for cellular tumor antigen p53 (TP53), the other 11 proteins have been shown to be upregulated in different types of cancer (Table S6).^33−45^ Six of these proteins are involved in the folding, aggregation, or turnover of proteins [hypoxia up-regulated protein 1 (HYOU1), heat shock 70 kDa protein 1A or 1B (HSPA1A or 1B), protein disulfide-isomerase (P4HB), prolyl 4-hydroxylase subunit alpha-2 (P4HA2), serpin H1 (SERPINH1), ubiquitin carboxyl-terminal hydrolase isozyme L1 (UCHL1)]. Two are involved in l-serine biosynthesis [phosphoserine aminotransferase (PSAT1), phosphoserine phosphatase (PSPH)]. Three are involved in mRNA transcription or in posttranscriptional gene silencing [TP53, signal transducer and activator of transcription 3 (STAT3), probable ATP-dependent RNA helicase DDX5 (DDX5)]. One is involved in double-strand break repair and regulation of protein phosphorylation [182 kDa tankyrase-1-binding protein (TNKS1BP1)]. Downregulation of the abundance of this set of proteins by NR exposure may have a role in the delayed cell growth and induction of cell death observed in the present work.

### NR Cytotoxicity to BEAS-2B Cell Spheroids and Alteration of Energy Status

**Figure 12:**
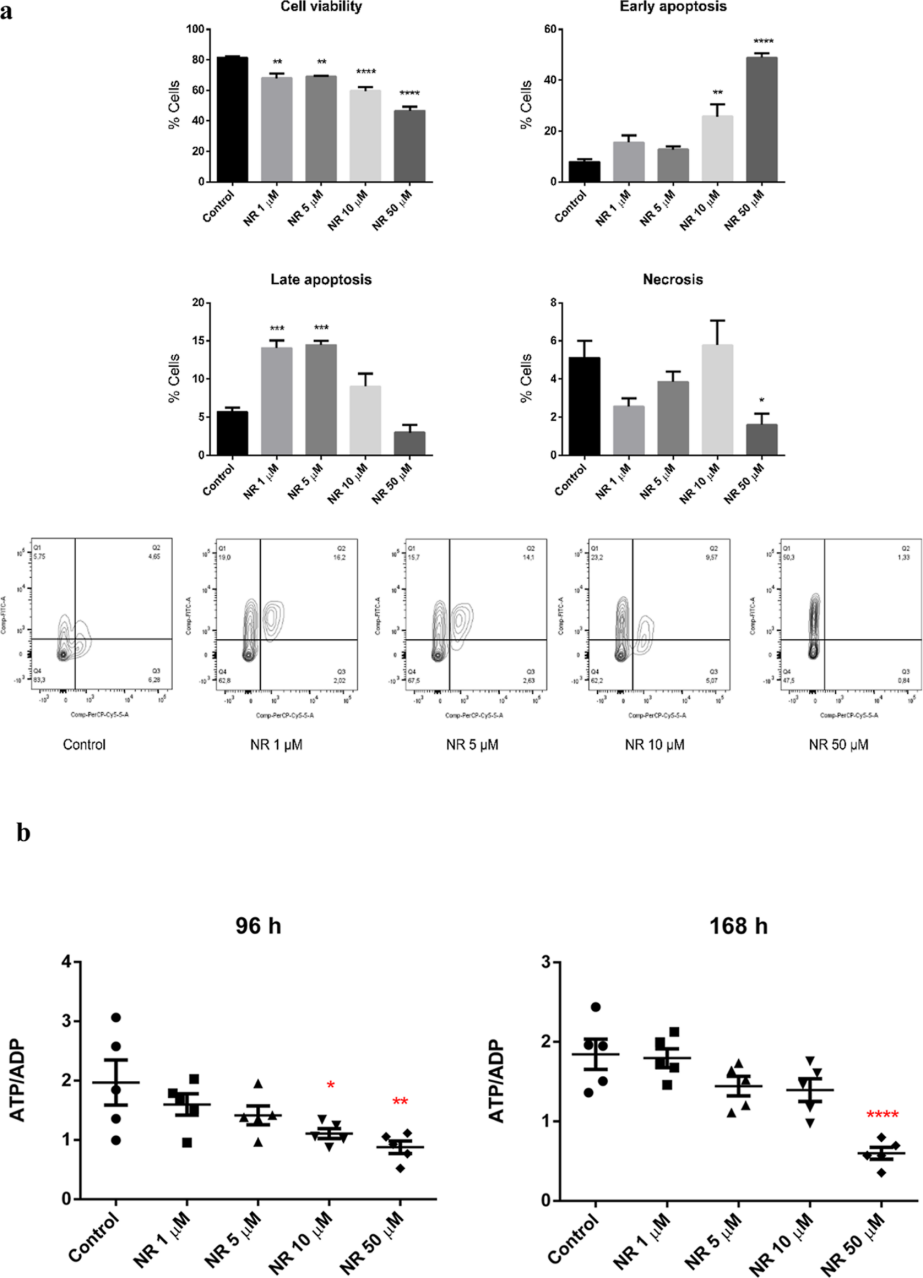
NR chloride (NR) induced (a) apoptosis and (b) energy stress in BEAS-2B cell spheroids. The spheroids were exposed daily to the indicated concentrations of NR chloride. The viability and type of cell death were assessed after 168 h of exposure. Q1, early apoptosis; Q2, late apoptosis; Q3, necrosis; Q4, viable cells. The ATP/ADP ratio was assessed after 96 and 168 h of exposure. Statistics of (a): one-way ANOVA with Dunnett’s multiple comparisons test, *N* = 4 (control, NR 1, 5, 10 μM) or *N* = 3 (NR 50 μM). Statistics of (b): one-way ANOVA with Dunnett’s multiple comparisons test, *N* = 5. *p < 0.05, **p < 0.01, ***p < 0.001, ****p < 0.0001.

Given the NR cytotoxicity to the BEAS-2B cells grown in a monolayer, we investigated if the culture condition could affect the viability of the cells exposed to NR. The BEAS-2B cells were then grown in spheroids (3D cell culture model) and exposed daily to 1, 5, 10, and 50 μM NAM riboside chloride (Sigma-Aldrich, Cat. number SMB00907, CAS number 23111-00-4) for 168 h. The cell culture medium used for the spheroids growth and exposure was the LHC-9 Medium from Gibco (Catalog number: 12680013), instead of the bronchial epithelial cell growth basal medium (BEBM) with supplements and growth factors provided by Lonza (catalog no. CC-3170) used for the 2D cell culture model. As seen in Figure 12a, NR chloride induced apoptosis of spheroid cells at all concentrations tested. However, the cells exposed to 1 and 5 μM of NR chloride were mainly in late apoptosis, while those exposed to 10 and 50 μM were in early apoptosis at 168 h.

We explored the energy status of the spheroid cells by ATP and ADP quantification at 96 and 168 h of NR chloride exposure. The ATP/ADP ratio calculated for each spheroid sample revealed that 10 and 50 μM NR chloride induced energy stress (Figure 12b).

### Comparative Cytotoxicity of NAM Riboside in the BEAS-2B and 1198 Cell Lines Grown in a Monolayer

Cancer development involves the reprogramming of cell energy metabolism.^46^ Therefore, the modulation of cell energy metabolism by NR may differentially affect the survival of normal and premalignant cell lines. We assessed this possibility by comparing the cytotoxicity induced by NR in the BEAS-2B human cell line and its premalignant derivative, the 1198 human cell line.

**Figure 13:**
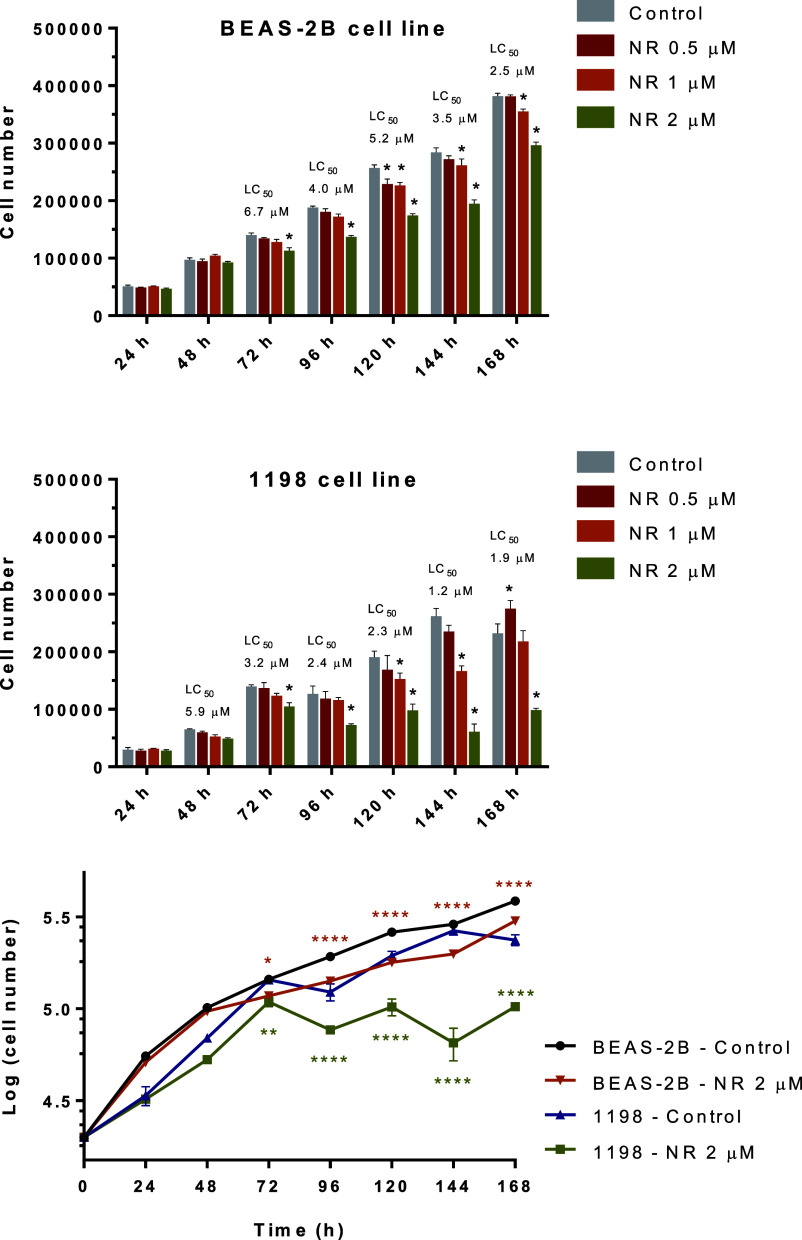
NR is preferentially toxic to the premalignant 1198 cell line in the cultures grown in a monolayer. The first two panels show the cell number obtained by the CVD assay for each NR concentration and time point of daily exposure. The calculated lethal concentration for 50% of the cells (LC_50_) is shown above each time point. The last panel shows the growth curves of BEAS-2B and 1198 cell lines exposed or not to 2 μM NR. Asterisks indicate the significant differences between the exposed and control cells of each cell line. First two panels’ statistics: two-way ANOVA with Dunnett’s multiple comparisons test, *N* = 4 or 5. Last panel’s statistics: two-way ANOVA with Tukey’s multiple comparisons test, *N* = 5, *p < 0.05, **p < 0.01, ****p < 0.0001.

Cytotoxicity (through CVD assay) was assessed every 24 h over 168 h of exposure to NR in the concentration range from 0.5 to 2 μM. As shown in Figure 13, the premalignant 1198 cells were more sensitive than the BEAS-2B cells to the toxic effect of NR.

## Discussion

This is the first report of systematic monitoring of cell survival and energy status after repeated in vitro cell exposure to NR. In contrast to those of other studies,^1,12^ the data presented here show that NR is capable of inducing cytotoxicity at the low μM range under the described culture conditions of BEAS-2B cells grown in a monolayer or in spheroids (Figures 3 and 12a).

A peculiarity of the present study was the use of a serum-free cell culture medium—the BEBM—with supplements and growth factors provided by Lonza (catalog no. CC-3170) or the LHC-9 medium from Gibco (catalog number: 12680013). BEBM is a proprietary modification of Laboratory of Human Carcinogenesis basal medium #9 (LHC-9), consisting of the primary formulation.^29,47^ The NAD^+^ precursor in BEBM and LHC-9 medium that can be used by human bronchial epithelial cells is NAM, as the lung does not have the complete set of enzymes for *de novo* NAD^+^ synthesis from tryptophan.^28^ An important difference between the culture media used here and the culture media used in other studies that have investigated the in vitro effects of NR is the concentration of NAM. The widely used Dulbecco’s modified Eagle’s medium (DMEM) contains 32.7 μM NAM,^28^ while BEBM or LHC-9 medium contain 0.3 μM NAM.^29^ The addition of 1 μM NR to BEBM or LHC-9 medium represents a 4.3-fold increase in the availability of a NAD^+^ precursor to the cells. For comparison, the concentration of NAM in mouse serum is 2 μM,^28^ and in human plasma, it is approximately 4 μM.^48^

The addition of 1 μM NR to BEAS-2B cells grown in a monolayer induced the expression of two genes that encode enzymes that catalyze the phosphoribosylation and phosphorylation of NAM and NR, respectively: *NAMPT* and *NMRK1* (Figure 7). These enzymes catalyze the reactions in the first step of the NAD^+^ synthesis pathways from each precursor (Figure 1). As a fraction of NR is converted to NAM in the culture medium,^49^ the NR-exposed cells were also exposed to a higher concentration of NAM than the control cells. The estimated half-life of NR in the cell-free culture medium was approximately 20 h (Figure 2c). The data provide evidence that BEAS-2B cells adapted their NAD^+^ metabolome in the presence of NR as an additional NAD^+^ precursor. The persistent exposure to NR led to the preferential upregulation of the *NMRK1* gene expression, which may have increased the ability of the cells to convert NR to NAM mononucleotide for NAD^+^ synthesis (Figure 1). This outcome may have improved the capability of the cells to increase the NAD^+^ content over time, as the expression of NMRK is rate-limiting for NR conversion.^49^ As NAMPT is subject to feedback inhibition by NAD^+^,^50^ the increased expression of *NMRK1* may have contributed to sustained NAD^+^ synthesis.

Human cells also express the NMRK2 enzyme, which is 57% identical to NMRK1 and catalyzes the same reaction of NR phosphorylation to NAM mononucleotide.^2^ There is evidence that *Nmrk2* gene expression is induced by energy stress in cultured rat cardiomyocytes via AMP-activated protein kinase/peroxisome proliferator-activated receptor α (AMPK-PPARα) activation.^32^ On the other hand, *Nmrk1* gene expression did not change under the conditions that led to energy stress in cultured rat cardiomyocytes or in the heart of a mouse model of dilated cardiomyopathy.^32^ We did not include the *NMRK2* gene expression analysis in the present study because it is primarily restricted to muscle.^51^ The role of cellular energy stress in inducing *NMRK1* gene expression needs further investigation.

According to the changes observed in *NAMPT* and *NMRK1* expression, the NAD^+^ content of BEAS-2B cells exposed to 1 μM NR increased 1.5-fold compared to that of the control at 192 h (control: 5.5 ± 0.1; NR: 8.1 ± 0.6 pmol/μg protein). Smaller increases that were not significant were noticed at other time points, and a decrease occurred at 144 h (Figure 5). The 1.5-fold increase in NAD^+^ at 192 h approximates the increase obtained after the exposure of different cell lines to much higher concentrations of NR (500 μM to 1 mM) for 24 or 48 h.^1,11^ The quantified levels of NAD^+^ in the BEAS-2B cells were on the same order of magnitude as those observed in other studies of other cell types.^49,52^

An early effect of NR in BEAS-2B cells grown in a monolayer was the decrease in the ATP/ADP and ATP/AMP ratios, indicative of the energy stress that persisted in the exposure range from 72 to 168 h (Figure 6). Spheroids of BEAS-2B cells exposed to 10 and 50 μM NR chloride for 96 and 168 h also showed a decreased ATP/ADP ratio (Figure 12b). This was an unexpected effect, given the known role of NR in the enhancement of mitochondrial function.^11^ However, as pointed out in certain studies, the link between an increase in NAD^+^ content induced by NR and the enhancement of mitochondrial respiration parameters is not obvious.^11,32^ Cantó and co-workers (2012) mentioned that the “increase in NAD^+^ was not linked to changes in cellular glycolytic rates or ATP levels (data not shown)”, referring to the effects of NR in mammalian cells in vitro and in mouse tissues.^11^ Diguet and co-workers (2018) verified that NR (100, 250, 500, and 1000 μM) did not alter the mitochondrial respiration parameters of neonatal rat cardiomyocytes in vitro. Despite the increase in NAD^+^ content, NR was not capable of attenuating the impact of the Nampt inhibitor FK866 on cell metabolism, for example, the decrease of basal oxygen consumption rates, maximal respiration, and ATP production, further reducing maximal respiration at high doses. However, glycolysis was stimulated.^32^ Moreover, healthy subjects who received NR (1000 mg/day) over 6 weeks presented with increased mean levels of AMP (2.9-fold), ADP (2.0-fold), and ATP (1.4-fold) in circulating PBMCs compared to the placebo group; however, these levels did not differ statistically. The ATP/ADP and ATP/AMP ratios tended to decrease in the NR-supplemented group.^17^

Proteomic analysis of monolayer-cultured BEAS-2B cells exposed to 1 μM NR for 144 h pointed to glycolysis stimulation for ATP synthesis, increased generation and use of cytosolic NADH for energy production, increased antioxidant activity and fatty acid synthesis, modulation of amino acid metabolism, as well as increased ribose and deoxyribose phosphate generation for nucleotide and nucleic acid synthesis (Figures 9b, 10, S3, S4, Table S4). We observed upregulation of several oxidoreductases in NR-exposed cells, which is consistent with the increased MTT reducing capacity shown in Figure 3b,c. In parallel, NR exposure led to decreased abundance of proteins involved in the folding, aggregation, or turnover of proteins, in l-serine biosynthesis, mRNA transcription, posttranscriptional gene silencing, double-strand break repair, and regulation of protein phosphorylation (Figures 9c, 11, S5 and Table S6). Among the 12 downregulated proteins described here, 11 have been shown to be upregulated in different types of cancer.^33−45^ Downregulation of these proteins may have a role in slowing the growth and inducing apoptosis of the NR exposed cells, despite the shift to glycolytic metabolism. To the best of our knowledge, this is the first report showing the modulation of the abundance of the described sets of proteins by NR exposure.

Interestingly, BEAS-2B cells showed rescued energy homeostasis after 192 h of exposure to 1 μM NR (Figure 6a, b). This amelioration was accompanied by an increase in NAD^+^ content (Figure 5), the return of *NMRK1* gene expression to the control level (Figure 7), and the normalization of the cell cycle (Figure 4a) and cell growth (Figure 3a). Notably, the cells grew throughout the total experimental period but started to reach a plateau in the 144–192 h interval (Figure 3d). Therefore, NR induced energy stress during the exponential phase of cell growth when the ATP request was high. Once the growth rate was slowed, cellular energy consumption decreased, which may have favored energy homeostasis and NAD^+^ synthesis. As observed in the correlation analyses during the 72–192 h incubation period (Figure 6c), the changes in NAD^+^ seem to be interconnected with the changes in AMP, ADP, and ATP levels induced by NR.

The mechanisms behind the induction of energy stress by NR and cellular metabolic reprogramming deserve investigation.

It was verified that Nmrk1-knockout mice displayed altered hepatic metabolism and function. The deficiency in Nmrk1 affected mitochondrial respiration through complex I + II and maximal electron transport system capacity, impairing gluconeogenesis. When liver-specific Nmrk1-knockout mice were fed a high-fat diet, liver damage clearly occurred. These mice were not given supplemental NR, but an approximate 2-fold increase in hepatic NR content was observed.^53^ The toxicity of persistently elevated hepatic NR was not considered by Sambeat and co-workers,^53^ as this effect was not known. However, the impaired mitochondrial respiration in the Nmrk1-knockout mice is in line with our observations of energy stress and decreased mitochondrial respiration induced by NR in BEAS-2B cells (Figure 8).

Of note, NR exposure for 144 h increased the abundance of PNP in BEAS-2B cells (Figure 10 and Table S4). PNP catalyzes the phosphorolysis of purine nucleosides, with the formation of the corresponding free purine base and ribose-1-phosphate. It is involved in purine metabolism, nucleotide metabolism, and nicotinate and NAM metabolism.^54−56^ In NAM metabolism, PNP catalyzes the reversible reaction of NR phosphorolysis to NAM and ribose-1-phosphate. It has been shown that this is the main pathway of NR metabolism in mammalian cells, and the bioavailability of NR for NMN synthesis through NMRK increased after PNP downregulation or inhibition, although not always leading to increased cellular NAD^+^ content.^57^ Strategies to improve the stability of NR against the action of PNP are underway to improve the bioavailability of NR and maximize its beneficial effects.^58^

NAM resulting from NR phosphorolysis can enter the salvage pathway for NAD^+^ synthesis. However, ribose-1-phosphate is often overlooked as a degradation product of NR. Once ribose-1-phosphate is converted to fructose-6-phosphate and glyceraldehyde-3-phosphate through a few steps of the pentose phosphate pathway, it can fuel glycolysis. It is also converted into ribose-5-phosphate and 5-phosphoribosyl-1-pyrophosphate, needed for purine, pyrimidine, and histidine metabolism, as well as for NAM phosphoribosylation to NMN.^54^ Thus, NR would induce effects that go beyond the effects due to NAD^+^ generation. The proteomic analysis shown here points to the increase of glycolysis and gluconeogenesis, amino acid metabolism, 2′-deoxyribonucleotide metabolic process, monosaccharide biosynthetic process, and superoxide dismutase activity due to NR exposure.

Uridine phosphorylase UPP1/UPP2 catalyzes the phosphorolysis of uridine into uracil and ribose-1-phosphate. It was shown that uridine-derived ribose-1-phosphate is converted to fructose-6-phosphate and glyceraldehyde-3-phosphate and fuel glycolysis, ATP production, biosynthesis, and gluconeogenesis in several cell lines, macrophages, and mice, remarkably when glucose is limiting.^59^ It was also shown that uridine-derived ribose-1-phosphate can enter glycolysis in a constitutive way, in contrast to glucose, by bypassing regulatory steps of upper glycolysis. So, ribose-1-phosphate can fuel glycolysis even in the presence of high glucose levels.^59^

The exposure of pancreatic cell lines to ribose-labeled [^13^C_5_]uridine, to trace the metabolic fate of ribose-1-phosphate, revealed, besides UMP and UTP labeling, ATP, AMP, ADP (all M + 5), and NAD^+^ (M + 5, M + 10) labeling, which shows the use of ribose-1-phosphate for ribosylation of adenine and NAM. Also labeled were glycolysis (phosphoenolpyruvate, pyruvate, lactate), pentose phosphate pathway (xylulose-5-phosphate and ribose-5-phosphate), hexosamine biosynthetic pathway (uridine diphosphate *N*-acetylglucosamine), tricarboxylic acid cycle intermediates (malate and citrate), nonessential amino acids (aspartate, glutamate, and serine), and oxidized glutathione.^60^

Thus, NR phosphorolysis by PNP may be a way in which NR increases NAD^+^ generation and metabolic effects above those provided by a same dose of NAM. The role of ribose-1-phosphate in NR effects deserves further investigation.

Additionally, it is known that NR elicits a rapid increase in mitochondrial NAD^+^ levels, which was observed in HEK293T cells.^11^ Mitochondrial NAD^+^ activates SIRT3, which regulates the β-oxidation of fatty acids,^11,61^ a source of AMP.

Energy and nutrient stress situations, such as exercise, fasting, and calorie restriction, activate the β-oxidation of fatty acids.^61,62^ Under these situations, mitochondrial oxidative phosphorylation becomes the main source of cellular ATP, whose production and hydrolysis to ADP are tightly regulated to maintain proper mitochondrial function and cell survival.^63−65^ While the decrease in the ATP/ADP ratio favors mitochondrial respiration, the increase in AMP activates AMPK. Activated AMPK induces the expression of *NAMPT* and *NMRK2* with subsequent NAD^+^ synthesis. It also contributes to the activation of transcription factors such as PGC1α and FOXO, triggering the modulation of pathways that result in mitochondrial biogenesis and regulation of diverse cellular processes, such as apoptosis, cell cycle, redox stress, and oxidative metabolism, with health benefits such as cancer prevention.^32,62,66^

As shown here, the premalignant 1198 cell line, which was derived from tumors originating from BEAS-2B cells exposed to cigarette smoke condensate transfected into mice,^30^ was more sensitive than the parent normal cell line to the cytotoxic effect of NR (Figure 13). NR at a concentration of 2 μM delayed the growth of BEAS-2B cells, but they were still growing during the exposure period. However, 2 μM NR induced the growth arrest of 1198 cells at 72 h, which persisted until the 168th hour. This is the first observation of NR cytotoxicity preferentially induced in a premalignant cell line.

The systemic oral bioavailability of NR is considered extremely low due to the almost complete first pass metabolism of NR to NAM and NAD^+^.^28^ In fact, it is difficult to detect an increase in NR concentration in human whole blood even after supplementation with high NR doses over several days.^16,27^ However, there are many benefits of NR supplementation reported in animal experimental models.^11,18−26^ Although the benefits are attributed to increased NAD^+^ levels, the present study points to an effect of NR on energy metabolism and modulation of cell proteome. More studies are necessary to understand the role of the energy stress induced by NR in its beneficial effects. There is also a need to assess whether the changes induced by NR give rise to a new normal cell phenotype, which may respond better to stress conditions.

BEAS-2B cells appear to be an interesting model to reveal effects due to NR exposure that have not been reported in another in vitro experimental model.

Finally, we highlight that some factors that facilitated the observations presented here were: (1) the use of a serum-free cell culture medium with a very low concentration of NAM, (2) the daily change of NR-supplemented medium, and (3) monitoring the effects over different growth phases of the cell culture. New in vitro studies aimed at understanding the mechanisms of action of NR should take these factors into account.

## Methods

### Chemicals and Enzymes

All the chemicals employed here were of the highest purity grade commercially available. Chromatography-grade acetonitrile and methanol were obtained from Carlo Erba Reagents (Milan, Italy). Sodium hydroxide, potassium phosphate, and ammonium acetate were acquired from Merck (Darmstadt, Germany). The Bradford reagent for protein quantification was obtained from BioRad (Hercules, CA, USA). Trypsin/ethylenediaminetetraacetate (EDTA) solution was from Vitrocell (Campinas, SP, Brazil). Unless specified, all the other reagents were obtained from Sigma-Aldrich Co. (St. Louis, MO). Water was purified in a Milli-Q system (Millipore, Bedford, MA).

### NAM Riboside

NR was obtained from the alkaline phosphatase hydrolysis of NAM mononucleotide, as previously reported.^2^ NMN (15 mg) was incubated with 90 units of alkaline phosphatase for 3 h at 37 °C in 3 mL of buffer (100 mM NaCl, 20 mM Tris, 5 mM MgCl_2_, pH 8.0). The hydrolysis product was purified by HPLC-PDA (Shimadzu, Kyoto, Japan). The chromatographic condition consisted of a 250 × 4.6 mm i.d., 5 μm, Shim-pack VP-ODS column (Shimadzu, Kyoto, Japan) with a C18 4.0 × 3.0 mm precolumn (Phenomenex, Torrance, CA) that was eluted with a gradient of water (solution A) and methanol (solution B) at a flow rate of 1 mL/min and 30 °C as follows: 0–5 min, 0% B; 5–20 min, 0–50% B. NR was detected at λ = 266 nm and eluted in the 4–6 min interval. The chromatographic fraction containing NR was collected, lyophilized, and reanalyzed for purity assessment. The NR aqueous solution was centrifuged through a 3000 Da filter (Amicon Ultra, Merck Millipore) for sterilization and removal of any protein residue. NR concentration in the aqueous stock solution was determined using the molar extinction coefficient of 5700 M^–1^ cm^–1^ at 266 nm.^67^ The identity of NR was confirmed by its mass spectra, which were obtained by the injection of the pure solution diluted in methanol into a maXis 3G QTOF mass spectrometer (Bruker Daltonics, Bremen, Germany) with electrospray ionization in positive mode. The following conditions were used: end plate offset, 500 V; capillary, 4500 V; nebulizer, 0.8 bar; dry gas, 4 L/min; temperature, 200 °C; collision energy in the MS2 spectrum, 10 eV.

### NAM Riboside Stability

The frozen (−80 °C) aqueous stock solution of NR was monitored spectrophotometrically (λ = 266 nm) at days 1, 15, 45, 75, and 90 for stability assessment. Additionally, NR stability in the cell-free culture medium at 37 °C was monitored over 24 h, starting with a solution of 44 μM NR. Aliquots were collected at 1, 3, 5, 7, 16, 20, and 24 h, and 20 μL was injected into the HPLC-PDA system (Shimadzu, Kyoto, Japan) for NR quantification (λ = 266 nm). The chromatographic condition consisted of a 250 × 4.6 mm i.d., 5 μm, Shim-pack VP-ODS column (Shimadzu, Kyoto, Japan) with a C18 4.0 × 3.0 mm precolumn (Phenomenex, Torrance, CA) that was eluted with a gradient of 0.1% formic acid in water (solution A) and methanol (solution B) at a flow rate of 1 mL/min and 30 °C as follows: 0–5 min, 0% B; 5–10 min, 0–100% B; 10–20 min, 100–0% B. Analyses were in quadruplicate for each time point. A calibration curve of NR was prepared in the 10–50 μM range and 20 μL injections were performed.

### Cell Culture

The cell lines BEAS-2B and 1198 were kindly donated by Prof. Fekadu Kassie (University of Minnesota, Minneapolis, MN, USA). The phenotypically normal human bronchial epithelial cell line BEAS-2B was authenticated by the method of short tandem repeats (Banco de Células do Rio de Janeiro, RJ, Brazil). The detected profile was compatible with the BEAS-2B profile described by the American Type Culture Collection. Mycoplasma contamination was assessed periodically by conventional polymerase chain reaction (PCR) using the primers oligo sense (5′ GGC GAA TGG GTG AGT AAC ACG 3′) and oligo antisense (5′ CGG ATA ACG GTT GCG ACC TAT 3′). The amplified samples were submitted to electrophoresis (100 V, 300 mA) in 3% agarose gel. The gel was then incubated with 0.01% solution of Red Gel in Milli-Q water. The bands were detected by a transilluminator. Parallel positive controls were run.

The cell lines were cultured in BEBM with supplements and growth factors provided by Lonza (catalog no. CC-3170). Conventional culture conditions (37 °C, 5% CO_2_) were used, and the cells were subcultured at 80% confluence.

### BEAS-2B Cell Spheroid Culture and Exposure to NAM Riboside

The spheroids grew in 48-well plates precoated with a solution of 1.5% agarose in phosphate buffered saline (PBS). BEAS-2B cells suspended in LHC-9 Medium from Gibco (catalog number: 12680013) were distributed to the agarose-coated wells in the concentration of 2 × 10^4^ cells/well and incubated at 37 °C and 5% CO_2_. Under these conditions, 1 spheroid per well was obtained. Medium change occurred daily, starting from the second day of incubation. Exposure to NR chloride (Sigma-Aldrich, Cat. number SMB00907, CAS number 23111-00-4) began on the fifth day of spheroid growth. The spheroids were separated into groups according to the concentration of NR chloride they were exposed to (1, 5, 10, or 50 μM). The culture medium containing the corresponding concentration of NR chloride was changed every day and the exposure occurred over 168 h (7 days).

### CVD Assay

As the dead cells do not adhere to the culture plates, it is possible to assess cell growth and survival by staining the adhered cells with the CVD. Cells were plated (2 × 10^4^ cells/well) into 96-well plates and the exposure to NR was initiated after the period of 24 h of cell adhesion. After each exposure period, the culture medium was removed, and the cell layer was washed twice with PBS. Methanol (100 μL) was added to fix the cells and rapidly aspirated. The cells were stained with 50 μL/well of the CVD solution (1% CVD in 20% methanol) for 10 min at room temperature. The excess dye solution was removed, and the wells were washed 4 times with 200 μL of PBS. The cell-bound dye was dissolved with 200 μL of 30% acetic acid. Standard curves were constructed by plating the cells in the range from 0.5 × 10^4^ to 20 × 10^4^ cells/well. The absorbance at 570 nm, subtracting the plate absorbance at 690 nm, was recorded using a SpectraMax 190 plate reader (Molecular Devices, Sunnyvale, CA, USA) or a Synergy H1 plate reader (BioTek, Winooski, VT, USA). Cell images were obtained prior to the last step of the assay, using a Leica MC170 HD camera coupled to the inverted microscope Leica DMi1. The software Leica Application Suite V4 (LAS V4) was used for image acquisition.

### Cell Population Doubling Time Calculation

PDT was calculated using the cell number obtained in the CVD assay and the following equation: PDT = 1/*r*; *r* = 3.32 × (log Nh-log Ni)/(t2 – t1), in which *r* = cell multiplication rate, Nh = number of cells collected in a time point (t2), Ni = number of cells initially plated (t1), and 3.32 is a constant.

### MTT Assay

Cells were plated (2 × 10^4^ cells/well) into 96-well plates and the exposure to NR was initiated after the period of 24 h of cell adhesion. After each exposure period, the culture medium was removed, and the cell layer was washed with PBS. Culture medium containing 75 μL of the MTT solution (5 mg/mL in PBS) was added to the cells, followed by 2 h of incubation at 37 °C, 5% CO_2_. The culture medium was removed, and the blue precipitated formazan crystals were dissolved with 100 μL of DMSO. The absorbance at 595 nm, subtracting the plate absorbance at 690 nm, was recorded using a SpectraMax 190 plate reader (Molecular Devices, Sunnyvale, CA, USA). Data are presented as percentage relative to the control group.

### Cell Cycle Analysis

Cells were plated (4 × 10^4^ cells/well) into 24-well plates and the exposure to NR was initiated after the period of 24 h of cell adhesion. After each exposure period, the culture medium was removed, and the cell layer was washed with PBS. Tripsin/EDTA solution was used to suspend the cells. Phenol-red free DMEM with 2% FBS (1000 μL) was added and the cells were precipitated (500 g, 10 min, 4 °C) inside flow cytometry tubes. The cell pellet was suspended in 150 μL of fluorescent buffer (PBS, 2% FBS, 0.05% Triton X-100, 0.1% sodium citrate, 25 μg/mL propidium iodide) and 5 μL of RNase A solution (15 mg/mL). After 30 min at room temperature, the fluorescence intensity of 10,000 events per sample was monitored on the PerCP–Cy 5.5 channel (λ_exc_ = 488 nm, λ_emi_ = 695 nm) of the flow cytometer BD FACSCanto II (BD, Biosciences, EUA). Flowjo software (Flowjo LCC, Oregon, USA) was used for data analysis.

### Gene Expression

Cells were plated (3 × 10^5^ cells/well) into 6-well plates and the exposure to NR was initiated after the period of 24 h of cell adhesion. After each exposure period, the culture medium was removed, and the cell layer was washed three times with cold PBS. Total cell RNA was isolated using the RNeasy Mini Kit (Qiagen, Hilden, Germany), and its purity and integrity were confirmed using a Bioanalyzer 2100 system (Agilent Technologies, Santa Clara, CA, USA). Then, 2 μg of RNA was converted to cDNA using a High-capacity cDNA Reverse Transcription kit (Applied Biosystems, NJ, USA). For real-time PCR, the cDNA was diluted to 50 ng/μL, and forward and reverse primers, Taqman Gene Expression Master Mix (Life Technologies, Carlsbad, CA, USA), and RNase-free water were added to the cDNA mixture. The following primers obtained from Life Technologies were used: Hs00237184_m1 for *NAMPT*, Hs00944470_m1 for *NMRK1* and Hs01060665_g1 for *ACTB* (β-actin) as the endogenous control. The reaction was performed in an StepOnePlus-RT-PCR system (Applied Biosystems, NJ, USA) under the following conditions: 50 °C for 2 min, 95 °C for 10 min, 40 cycles of 15 s at 95, and 60 °C for 1 min. The gene expression levels were determined by comparing the C_T_ values for the genes of interest to those of the β-actin gene.

### NAD^+^, ATP, ADP, AMP, and Lactate Quantification in BEAS-2B Cells Grown in a Monolayer

Cells were plated (2 × 10^4^ cells/well) into 96-well plates and the exposure to NR was initiated after a period of 24 h of cell adhesion. After each exposure period, the culture medium was removed, and the cell layer was washed with PBS. Sample preparation was performed as described by Rahman and co-workers,^68^ with some modifications. Cell metabolism was stopped by the addition of 100 μL of cold extraction solution (90% acetonitrile and 10% ammonium acetate buffer 5 mM, pH 8.5). Samples were stored at −80 °C for 24 h, centrifuged (20,000*g*, 15 min, 4 °C), and the supernatant was collected and stored at −80 °C. The cell pellet was suspended in 385 μL of another cold extraction solution (40% acetonitrile, 40% methanol and 20% water) and 15 μL of the internal standard [^13^C_10_^15^N_5_]ATP (500 pmol), centrifuged (20,000*g*, 15 min, 4 °C), and the supernatant was combined with the first one. The volume of 450 μL was transferred to a new tube. After the addition of 3.02 μL of a 0.001 μg/μL solution of benzamide (25 pmol), used as the internal standard for NAD^+^ quantification, and 3.05 μL of a 0.01 μg/μL solution of benzoic acid (250 pmol), used as the internal standard for lactate quantification, the samples were vacuum-dried. The samples were resuspended in 30 μL of water and centrifuged at 16,000*g* for 5 min. The volume of 6 μL containing 90 pmol of the internal standard [^13^C_10_^15^N_5_]ATP, 5 pmol of the internal standard benzamide, and 50 pmol of the internal standard benzoic acid was injected into the HPLC-ESI-MS/MS system in negative and positive modes. The analytical system consisted of an Agilent 1200 series HPLC instrument (Wilmington, DE, USA) interfaced with a Linear Quadrupole Ion-Trap mass spectrometer (model 4000 QTRAP, Applied Biosystems/MDS Sciex Instruments, Foster City, CA, USA). The analyses were conducted using three methods. Method 1 was for AMP, ADP, and ATP quantification, with electrospray ionization in negative mode (ESI^–^, [M – H]^−^), employing the optimized parameters CUR, 18 psi; GS1, 40 psi; GS2, 45 psi; CAD, medium; TEM, 700 °C; EP, −10 V; IS, −4500 V. Method 2 was for NAD^+^ quantification, with electrospray ionization in positive mode (ESI^+^, [M + H]^+^), employing the optimized parameters CUR, 18 psi; GS1, 40 psi; GS2, 45 psi; CAD, medium; TEM, 700 °C; EP, 10 V; IS, 4500 V. Method 3 was for lactate quantification, with electrospray ionization in negative mode (ESI^–^, [M – H]^−^), employing the optimized parameters CUR, 18 psi; GS1, 40 psi; GS2, 45 psi; CAD, medium; TEM, 700 °C; EP, −10 V; IS, −4500 V.

The fragmentations and instrument parameters used in MRM mode are described in Table S7. The quantifications were based on the ratio of the peak area of each analyte to that of the internal standard ([^13^C_10_^15^N_5_]ATP for AMP, ADP and ATP quantification; benzamide for NAD^+^ quantification; benzoic acid for lactate quantification). The calibration curves (Figure S6) contained NAD^+^ (1.875–60 pmol), ATP (18.75–300 pmol), ADP (3.125–100 pmol), AMP (0.3125–10 pmol), and lactate (25–1600 pmol) as well as the internal standards [^13^C_10_^15^N_5_]ATP (90 pmol), benzamide (5 pmol), and benzoic acid (50 pmol) in the injection volume. The chromatographic conditions were as follows. Method 1: a Luna Omega PS C18 (150 × 2.1 mm i.d., 5.0 μm, 100A, Phenomenex) with a C18 4.0 × 3.0 mm precolumn (Phenomenex, Torrance, CA) was eluted with 0.01% acetic acid in water (solution A) and 40% acetonitrile in 10 mM ammonium acetate buffer (solution B) at 30 °C (0–2.5 min, 2–90% B, 400 μL/min; 2.5–3 min, 90–2% B, 400 μL/min; 3–6 min, 2% B, 400 μL/min; 6–6.5 min, 2–100% B, 400–600 μL/min; 6.5–9 min, 100% B, 600 μL/min; 9–9.5 min, 100–2% B, 600–400 μL/min; 9.5–15 min, 2% B, 400 μL/min). The chromatographic fraction between 2.5 and 8 min was directed to the mass spectrometer. Method 2: a Luna Omega PS C18 (150 × 2.1 mm i.d., 5.0 μm, 100A, Phenomenex) with a C18 4.0 × 3.0 mm precolumn (Phenomenex, Torrance, CA) was eluted with 0.01% formic acid in water (solution A) and 40% acetonitrile in 10 mM ammonium acetate buffer (solution B) at 30 °C (0–2.5 min, 0–90% B, 400 μL/min; 2.5–6 min, 90% B, 400 μL/min; 6–6.5 min, 90–2% B, 400–600 μL/min; 6.5–7 min, 2–90% B, 600 μL/min; 7–8 min, 90% B, 600 μL/min; 8–9 min, 90–0% B, 600 μL/min; 9–14 min, 0% B, 600 μL/min; 14–15 min, 0% B, 600–400 μL/min). The chromatographic fraction between 0.6 and 7 min was directed to the mass spectrometer. Method 3: a Luna Omega PS C18 (150 × 2.1 mm i.d., 5.0 μm, 100A, Phenomenex) with a C18 4.0 × 3.0 mm precolumn (Phenomenex, Torrance, CA) was eluted with 0.1% formic acid in water (solution A) and 40% acetonitrile in 10 mM ammonium acetate buffer (solution B) at 30 °C (0–4 min, 2–60% B, 450 μL/min; 4–4,5 min, 60–100% B, 450–600 μL/min; 4,5–5 min, 100% B, 600 μL/min; 5–5,5 min, 100–2% B, 600 μL/min; 5,5–6 min, 2–40% B, 600 μL/min; 6–6,5 min, 40–2% B, 600 μL/min; 6,5–14 min, 2% B, 600 μL/min; 14–15 min, 2% B, 600–450 μL/min). The chromatographic fraction between 0.5 and 8 min was directed to the mass spectrometer. The Analyst software, version 1.6 (Applied Biosystems/MDS Sciex Instruments), was used for data processing.

The precision of the methods was assessed by injections of a pooled sample containing 6 μL of each extracted sample. The coefficients of variation calculated as the relative standard deviation of each analyte amount (pmol) in six to eight analyses intercalated with the samples are shown in Table S8.

The cell pellet of each extraction solution was used for protein quantification by Bradford’s method.^69^ Proteins were dissolved in 40 μL of NaOH (300 mM) and stored at −20 °C. The solutions for quantification consisted of 195 μL of NaCl (150 mM), 5 μL of protein solution, and 50 μL of Bradford’s reagent. Calibration curves using bovine serum albumin were in the range from 0.0016 to 0.01 μg/μL.

### ATP and ADP Quantification in BEAS-2B Cell Spheroids

After each exposure period, the culture medium was removed and the spheroids were washed 2 times with PBS. The harvested spheroids were frozen in a solution of dry ice and ethanol, mechanically disrupted, and the cells were suspended in 100 μL of cold extraction solution (90% acetonitrile in water). After 24 h at −80 °C, 385 μL of another cold extraction solution (40% acetonitrile, 40% methanol and 20% water) and 15 μL of the internal standard [^13^C_10_^15^N_5_]ATP (500 pmol) were added. The suspensions were homogenized, centrifuged (20,000*g*, 15 min, 4 °C), and the supernatant was collected. The volume of 450 μL was transferred to a new tube and vacuum-dried. The samples were resuspended in 30 μL of water and centrifuged at 16,000*g* for 5 min. The volume of 6 μL containing 90 pmol of the internal standard [^13^C_10_^15^N_5_]ATP was injected into a Shimadzu UFLC system coupled to an ESI-Ion Trap mass spectrometer (amaZon Speed, Bruker Daltonics) in positive mode. The best parameters for each analyte detection and fragmentation were adjusted by direct infusion of the analytical standards into the mass spectrometer. Data were obtained in MRM mode and the following *m*/*z* transitions were used for quantification: *m*/*z* 523 → 425 ([^13^C_10_^15^N_5_]ATP), *m*/*z* 508 → 410 (ATP), *m*/*z* 428 → 136 (ADP). The chromatographic condition was as follows. Method 4: a Luna Omega PS C18 (150 × 2.1 mm i.d., 5.0 μm, 100A, Phenomenex) with a C18 4.0 × 3.0 mm precolumn (Phenomenex, Torrance, CA) was eluted with 0.01% formic acid in water (solution A) and 40% acetonitrile in 10 mM ammonium acetate buffer (solution B) at 30 °C and 700 μL/min (0–1 min, 0% B; 1–1.5 min, 0–100% B; 1.5–4 min, 100–30% B; 4–5 min, 30% B; 5–6 min, 30–20% B; 6–7 min, 20–0% B; 7–14 min, 0% B). The chromatographic fraction between 2 and 6.1 min was directed to the mass spectrometer. The software QuantAnalysis, version 4.3 (Bruker Daltonics), was used for data processing.

### Seahorse Analysis

The Seahorse XFe24 Extracellular Flux analyzer (Agilent Technologies, Santa Clara, CA, USA) was used for the analysis of mitochondrial respiration of BEAS-2B cells after 96 h of exposure to 1 μM NR chloride (Sigma-Aldrich, Cat. number SMB00907, CAS number 23111-00-4) compared to that of the control cells. Cells were plated (1 × 10^4^ cells/well) into 24-well plates (Seahorse XFe24 FluxPak mini) and the exposure to NR chloride was initiated after the period of 24 h of cell adhesion. After 96 h of exposure, the supernatant was removed, the cells were washed twice with 500 μL of prewarmed assay medium (RPMI medium without bicarbonate and FBS, pH 7.4), and the assay medium (400 μL) was added. Cells were left in a 37 °C incubator for 1 h. The injection conditions were: port-A, oligomycin (final concentration 1 μM); port-B and port-C, carbonyl cyanide 3-chlorophenylhydrazone (CCCP, final concentration 0.5 μM after each injection); port-D, rotenone + antimycin A (final concentration 1 μM). Oxygen consumption rate (OCR) was detected under basal conditions followed by the sequential addition of oligomycin, CCCP (two injections), and rotenone + antimycin A. Basal respiration, maximal respiration, spare respiratory capacity, proton leak, ATP production rate, coupling efficiency, and nonmitochondrial oxygen consumption were calculated using the Wave Desktop and Controller 2.6 software. Data were obtained with background correction and were normalized to the protein content in each well, determined by the Bradford’s method,^69^ as described above for NAD^+^, ATP, ADP, AMP, and lactate quantification. Negative OCR values were changed to zero for parameter calculations.

### Malondialdehyde Quantification

Cells were plated (2 × 10^4^ cells/well) into 96-well plates and the exposure to NR was initiated after the period of 24 h of cell adhesion. After each exposure period, a 100 μL aliquot of cell growth medium was used for malondialdehyde (MDA) quantification following a procedure described before.^70^ Protein-bound MDA was released by the addition of 10 μL of 4 M sodium hydroxide, and the mixture was incubated at 60 °C for 30 min under agitation (100 rpm). The proteins were then precipitated by the addition of 150 μL of 1% sulfuric acid, and the samples were centrifuged at 9300*g* for 10 min. Finally, 25 μL of dinitrophenylhydrazine (1 mg/mL in 2 M hydrochloric acid) was added to 175 μL of the supernatant, and the mixture was incubated at room temperature and protected from light for 30 min. Aliquots of 100 μL were injected into a HPLC-PDA system (Shimadzu, Kyoto, Japan). The chromatographic conditions used consisted of a 250 × 4.6 mm i.d., 5.0 μm, 100A Shim-pack VP-ODS column (Shimadzu, Kyoto, Japan) that was eluted with a gradient of water (solution A) and acetonitrile (solution B), both containing 0.2% acetic acid, at a flow rate of 1 mL/min and 30 °C as follows: 0–28 min, 20–100% B; 28–30 min, 100–20% B; and 30–40 min, 20% B. The DAD detector was fixed at 306 nm to detect the MDA-DNPH product. The calibration curves were in the MDA range of 0.05–6 μM.

The cell layer of each cell culture medium aliquot was used for protein quantification by the Bradford’s method,^69^ as described in the NAD^+^, ATP, ADP, AMP, and lactate quantification item.

### BEAS-2B Cell Spheroid Viability and Type of Cell Death Analysis

After the exposure period, 3 to 4 spheroids from the same group were transferred to a 5 mL glass tube and washed 2 times with PBS buffer. Tripsin/EDTA solution was used to suspend the cells. Once complete detachment of the cells from the spheroids was achieved, 1.2 mL of blocking solution (LHC-9 Medium from Gibco: FBS, 9:1) was added. The samples were centrifuged (769*g*, 5 min, 4 °C), the supernatant was removed, and the cells were washed 2 times with chilled PBS buffer. Staining with FITC Annexin V and propidium iodide was performed according to the manufacturer’s instructions (FITC Annexin V apoptosis detection Kit I, BD Pharmingen). The cells were suspended in 36 μL of assay buffer solution (10× Annexin V Binding Buffer) previously diluted in Milli-Q water (1:10), 2 μL of FITC Annexin V, and 2 μL of propidium iodide. The samples were incubated and protected from light for 20 min, and then 160 μL of prediluted assay buffer was added. The analyses were performed on the flow cytometer BD FACSCanto II (BD, Biosciences, EUA), where fluorescence intensities were monitored for 20,000 events. Flowjo software (Flowjo LCC, Oregon, USA) was used for data analysis.

### Sample Preparation for Proteomics

Cells were plated (4 × 10^5^ cells/well) into 6-well plates and the exposure to NR was initiated after the period of 24 h of cell adhesion. After 144 h of daily exposure to 1 μM NR, the culture medium was removed, and the cell layer was washed 5 times with ice-cold PBS. 500 μL of lysis buffer (Ambic 100 mM, urea 8 M and cOmplete protease inhibitor) was added to each well, cells were scraped, and the solution was transferred to a 2 mL tube and left on ice for 1 h. 12 U of benzonase was added with subsequent incubation for 10 min at 25 °C at 300 rpm. A new aliquot of 13 U of benzonase was added and incubated. The tubes were centrifuged (15 min, 15,000*g*, 4 °C) and the supernatant was transferred to a new tube. 1500 μL of ice-cold acetone (−20 °C) was added, and the samples were kept at −20 °C overnight for protein precipitation. After centrifugation (15,000*g*, 30 min, 4 °C), the supernatant was removed, the tubes were kept open for 5 min to dry the acetone, and 200 μL of resuspension buffer (100 mM Ambic, 8 M urea, 0.1% sodium deoxycholate) was added. Proteins were quantified following the Pierce BCA Protein Assay kit protocol from Thermo Scientific. 10 μg of proteins from each sample in 50 μL of buffer (Ambic 100 mM, urea 8 M) was reduced with 10 mM DTT for 1 h at 30 °C, alkylated with 15 mM iodoacetamide for 30 min in the dark at 25 °C, and the excess of iodoacetamide was quenched with 4.4 mM DTT for 15 min at 25 °C. Next, 450 μL of Ambic and 2.5 μL of trypsin (1:40 w/w) were added and the pH was checked to make sure it was above 7.0. The samples were incubated for 4 h at 30 °C and a new 2 μL aliquot of trypsin (1:50 w/w) was added. The pH was checked, and then overnight incubation at 30 °C was performed. Digestion was stopped with the addition of 200 μL of 4% trifluoroacetic acid. After 30 min at 37 °C, the tubes were centrifuged (14,000 rpm, 30 min, 4 °C), the supernatant was transferred to a new tube, and the samples were concentrated to the volume of 200 μL. Samples were desalted using the stage tip protocol,^71^ lyophilized, and stored at −80 °C until the injection into the mass spectrometer. Before injection, each sample was resuspended in 100 μL of formic acid 0.1%.^72^

### LC–MS/MS Measurements for Proteomics

The peptides were separated and analyzed in a Nano EASY-nLC 1200 (Thermo Fisher Scientific, Bremen, Germany) coupled to an Orbitrap Fusion Lumos mass spectrometer (Thermo Fisher Scientific, Bremen, Germany) as described before,^73^ with some modifications. Each sample (200 ng) was injected into a trap column (nano Viper C18, 3 μm, 75 μm × 2 cm, Thermo Scientific) with 20 μL of solvent A (0.1% formic acid) at 500 bar. Then, the peptides were eluted onto a C18 column (nano Viper C18, 2 μm, 75 μm × 15 cm, Thermo Scientific) at a flow rate of 300 nL/min. Peptides were eluted from the column using a linear gradient of solvent A (0.1% formic acid in water) and solvent B (0.1% formic acid in 20:80 water: acetonitrile, v/v), starting with 5–22% B in 55 min, followed by an increase to 32% B in 5 min. Next, column wash was accomplished with an increase of solvent B to 99% in 2 min, followed by 6 min with this solvent proportion. Re-equilibration of the system with 100% solvent A was performed before each injection. After ionization under positive electrospray conditions, the eluted peptides were analyzed in data-dependent acquisition mode. The most intense ions detected after a full scan (400–1600 *m*/*z*) at a 120,000 resolution were filtered for fragmentation by the quadrupole with a transmission window of 1.2 *m*/*z*, followed by HCD fragmentation with a normalized collision energy of 30, and detection of the fragments by the Orbitrap mass analyzer with a 30,000 resolution. A new cycle of MS followed by MS2 events occurred at every 3 s. Monocharged ions or ions with undetermined charges were excluded from fragmentation.

### Proteomics Data Analysis

The procedure was the same described before,^72^ except for the pathway enrichment analyses. Raw files were processed using MaxQuant.^74^ The Andromeda algorithm^75^ was used for protein identification against the *Homo sapiens* Uniprot database (downloaded August, 2019; 20416 entries). Error mass tolerance for precursors and fragments were set to 4, 5 ppm and 0,5 Da, respectively. Cysteine carbamidomethylation was selected as a fixed modification and methionine oxidation and *N*-terminal acetylation were selected as variable modification. Trypsin was set as digestion enzyme, with a maximum of 2 missed cleavages allowed. A maximum FDR of 1% was allowed for both peptides and proteins identification, and for proteins it was calculated using a decoy database created from the reverse ordination of the protein sequences in the Uniprot database. Identification of at least two peptides (unique + razor) was set as a parameter for the identification of a protein. Protein abundances were quantified by the LFQ algorithm, based on the normalized chromatographic peak integrations generated by MaxQuant. Other parameters were kept as default.

Before statistical analysis, the data were log-transformed, and matches to the contaminants and reverse database, as well as proteins identified only by modified sites, and missing values were filtered out. Statistical significance was assessed using a two-tailed Student’s *t*-test in the Perseus software^76^ (version 2.0.11) with a permutation-based FDR of 5% and a *S*_0_ parameter of 0.1.

For pathway enrichment analyses, we used the ClueGO^77^ app (version 2.5.10) in Cytoscape^78^ (version 3.10.1). ClueGO presents enrichment analysis as a network of interconnected nodes representing gene ontology (GO) biological processes, with edges representing kappa scores (a statistic based on the number of genes shared between different biological processes).^77^ Functional analysis was done selecting the ontologies/pathways WikiPathways, REACTOME_Pathways, GO_BiologicalProcess, using medium network specificity (GO tree interval 3–8) with 2 minimum number of genes and minimum 2 or 5% genes in the GO term/pathway selection, without GO term fusion, and with kappa score threshold 0.4 (medium). Bonferroni step down correction was applied to the results of a two-sided hypergeometric test. Only the processes with a corrected p value lower than 0.05 were visualized. GO term grouping was used. The leading group term was based on the highest significance result of kappa statistics. Redundant groups with >50% overlap were merged. The output tables of ClueGO enrichment analyses are provided in Tables S3 and S5.

### Statistics

The data are presented as mean ± SEM. The variables with normal distribution were compared among groups by a two-tailed *t*-test, one-way or two-way ANOVA with Tukey’s or Dunnett’s multiple comparisons test. Differences with *p* values < 0.05 were considered significant.

## Supporting Information Available

ATP, ADP, and AMP levels in BEAS-2B cells; detailed information on the heatmap clusters; output tables of ClueGO enrichment analyses; description of the proteins selected among the significant terms and pathways of the enrichment analyses; fragmentation and instrument parameters for the quantification of NAD^+^, ATP, ADP, AMP, and lactate in BEAS-2B cells; precision of the analytical methods for quantification of NAD^+^, ATP, ADP, AMP, and lactate in BEAS-2B cells; effect of the commercial NR chloride on the growth and morphology of BEAS-2B cells; correlation analyses of the levels of AMP, ADP, ATP, ATP/ADP, and ATP/AMP with the NAD^+^ content in the control cells; malondialdehyde and lactate levels in BEAS-2B cells; significant biological processes and pathways of ClueGO enrichment analyses; calibration curves for quantification of NAD^+^, ATP, ADP, AMP, and lactate in BEAS-2B cells (PDF)
(XLSX)

## Abbreviations

NR: nicotinamide riboside
NAD^+^: nicotinamide adenine dinucleotide
NADP^+^: nicotinamide adenine dinucleotide phosphate
NA: nicotinic acid
NAM: nicotinamide
NMN: nicotinamide mononucleotide
NaMN: nicotinic acid mononucleotide
NaR: nicotinic acid riboside
NaAD: nicotinic acid adenine dinucleotide
SIRT: sirtuin
NOAEL: no observed adverse effect level
HPLC-PDA: high-performance liquid chromatography coupled to photodiode array detector
CVD: crystal violet dye
MTT: yellow tetrazolium salt 3-(4,5-dimethylthiazol-2-yl)-2,5-diphenyltetrazolium bromide
ATP: adenosine triphosphate
ADP: adenosine diphosphate
AMP: adenosine monophosphate
NMRK1: nicotinamide riboside kinase 1
NAMPT: nicotinamide phosphoribosyltransferase
cDNA: complementary DNA
FDR: false discovery rate
BEBM: bronchial epithelial cell growth basal medium
LHC-9: Laboratory of Human Carcinogenesis basal medium #9
DMEM: Dulbecco’s modified Eagle’s medium
AMPK-PPARα: AMP-activated protein kinase/peroxisome proliferator-activated receptor α
PBMCs: peripheral blood mononuclear cells
UPP1/UPP2: uridine phosphorylase
UMP: uridine monophosphate
UTP: uridine triphosphate
PBS: phosphate buffered saline
PCR: polymerase chain reaction
PDT: cell population doubling time
DMSO: dimethyl sulfoxide
FBS: fetal bovine serum
OCR: oxygen consumption rate
CCCP: carbonyl cyanide 3-chlorophenylhydrazone
MDA: malondialdehyde

## Gene/protein symbols

NMRK1: nicotinamide riboside kinase 1
NAMPT: nicotinamide phosphoribosyltransferase
NQO1: NAD(P)H dehydrogenase [quinone] 1
SOD2: superoxide dismutase [Mn], mitochondrial
ALDH7A1: alpha-aminoadipic semialdehyde dehydrogenase
MDH2: malate dehydrogenase, mitochondrial
GOT1: aspartate aminotransferase, cytoplasmic
OAT: ornithine aminotransferase, mitochondrial
G6PD: glucose-6-phosphate 1-dehydrogenase
DUT: deoxyuridine 5′-triphosphate nucleotidohydrolase, mitochondrial
PNP: purine-nucleoside phosphorylase
RRM1: ribonucleoside-diphosphate reductase large subunit
ALDH18A1: delta-1-pyrroline-5-carboxylate synthase
ADH5: alcohol dehydrogenase class-3
PKM: pyruvate kinase PKM
ALDOA: fructose-bisphosphate aldolase A
ALDOC: fructose-bisphosphate aldolase C
ENO1: alpha-enolase
GPI: glucose-6-phosphate isomerase
PGK1: phosphoglycerate kinase 1
FASN: fatty acid synthase
HYOU1: hypoxia up-regulated protein 1
TP53: cellular tumor antigen p53
HSPA1A or HSPA1B: heat shock 70 kDa protein 1A or 1B
P4HB: protein disulfide-isomerase
STAT3: signal transducer and activator of transcription 3
P4HA2: prolyl 4-hydroxylase subunit alpha-2
SERPINH1: serpin H1 (HSP47)
PSAT1: phosphoserine aminotransferase
PSPH: phosphoserine phosphatase
TNKS1BP1: 182 kDa tankyrase-1-binding protein
UCHL1: ubiquitin carboxyl-terminal hydrolase isozyme L1
DDX5: probable ATP-dependent RNA helicase DDX5
