# Supplementary material for: Nicotinamide riboside Induced Energy Stress and Metabolic Reprogramming in BEAS-2B Cells
Source: Chem Res Toxicol. 2024 Jul 11;37(8):1246–68. doi: 10.1021/acs.chemrestox.3c00312 (PMC11337214; doi:10.1021/acs.chemrestox.3c00312)
Supplement: Supplementary file 1 — tx3c00312_si_001.pdf [file tx3c00312_si_001.pdf]

Supporting Information

Nicotinamide riboside induced energy stress and metabolic reprogramming in BEAS-2B cells

Everson Willian Fialho Cordeiro†, Elisabete Leide Marzola†, Ricardo Soei Maekawa†, Matheus Relvas dos Santos†, Lucas Gade Assunção†, Mariana Pereira Massafera‡, Joseana de Oliveira†, Thainá Gomes Cury Batista†, Maria Cármen Oliveira Pinho de Sales†, Silvya Stuchi Maria-Engler†, Paolo Di Mascio‡, Marisa Helena Gennari de Medeiros‡, Graziella Eliza Ronsein‡ and Ana Paula de Melo Loureiro†*

†Departamento de Análises Clínicas e Toxicológicas, Faculdade de Ciências Farmacêuticas, Universidade de São Paulo, Av. Prof. Lineu Prestes 580, CEP 05508-000, São Paulo, Brazil.

‡Departamento de Bioquímica, Instituto de Química, Universidade de São Paulo, Av. Prof. Lineu Prestes 748, CEP 05508-000, São Paulo, Brazil.

*Corresponding author: Phone: ++(55)1130911192, e-mail: apmlou@usp.br

Table of Contents

Table S1	page S3
Table S2	Excel file
Table S3	Excel file
Table S4	page S4 – S13
Table S5	Excel file

Table S6	page S14 – S20
Table S7	page S21
Table S8	page S22
Figure S1	page S23 – S24
Figure S2	page S25
Figure S3	page S26 – S27
Figure S4	page S28 – S29
Figure S5	page S30 – S31
Figure S6	page S32

Table S1. ATP, ADP and AMP levels (pmol/µg protein) in BEAS-2B cells exposed or not to nicotinamide riboside (NR) from 24 to 192 h. Asterisks indicate the significant difference between the NR exposed and control cells in each time point ($p < 0.05$). Unpaired t test with Welch's correction was used for the analyses.

ATP	Control (mean ± SEM)	NR (mean ± SEM)	ADP	Control (mean ± SEM)	NR (mean ± SEM)	AMP	Control (mean ± SEM)	NR (mean ± SEM)
25 h	19.2 ± 3.4, N=5	**35.6 ± 2.3***, N=5	25 h	3.4 ± 0.6, N=4	2.2 ± 0.2, N=5	25 h	1.1 ± 0.3, N=5	0.9 ± 0.1, N=5
27 h	16.6 ± 0.3, N=5	25.6 ± 3.4, N=5	27 h	2.2 ± 0.3, N=4	1.8 ± 0.2, N=5	27 h	1.7 ± 0.4, N=5	0.8 ± 0.1, N=5
72 h	31.9 ± 1.6, N=5	28.5 ± 1.4, N=5	72 h	3.58 ± 0.05, N=5	3.9 ± 0.2, N=5	72 h	0.6 ± 0.1, N=5	**1.3 ± 0.1***, N=5
96 h	36.0 ± 2.3, N=4	**27.8 ± 1.5***, N=4	96 h	3.3 ± 0.3, N=4	3.5 ± 0.2, N=4	96 h	0.32 ± 0.04, N=4	**0.50 ± 0.03***, N=4
120 h	42.9 ± 3.6, N=5	41.7 ± 2.0, N=4	120 h	4.6 ± 0.6, N=4	6.7 ± 0.7, N=4	120 h	0.5 ± 0.1, N=5	1.5 ± 0.4, N=4
144 h	37.2 ± 2.0, N=5	**22.8 ± 0.8***, N=5	144 h	4.2 ± 0.3, N=5	3.8 ± 0.2, N=5	144 h	0.28 ± 0.02, N=5	**0.51 ± 0.03***, N=5
168 h	32.5 ± 1.0, N=5	**26.7 ± 1.6***, N=5	168 h	4.2 ± 0.1, N=5	5.1 ± 0.3, N=5	168 h	0.46 ± 0.03, N=5	**0.89 ± 0.07***, N=5
192 h	29.8 ± 1.2, N=5	27.9 ± 1.1, N=5	192 h	6.6 ± 0.5, N=5	**4.7 ± 0.3***, N=5	192 h	1.6 ± 0.3, N=5	0.9 ± 0.1, N=4

Table S4. Nineteen proteins associated to 46 representative terms and pathways selected among the significant terms and pathways of **Table S3** and **Figure S4**). Selection criteria: at least 2 genes from the loaded list associated with a term (Number Genes) and representing at least 5% of the total number of genes in the term (% Associated Genes). The proteins belong to the cluster of 42 more abundant proteins in the NR group.

Gene symbol	Protein name	Protein function[a,b,c]	Associated terms
NQO1	NAD(P)H dehydrogenase [quinone] 1	Member of the NAD(P)H dehydrogenase (quinone) family. It catalyzes two-electron reduction of quinones to hydroquinones using either NADH or NADPH. It also scavenges superoxide radical. NQO1 gene expression may be induced by the generation of reactive oxygen species (ROS). Located in cytoplasm.<br>Reaction:<br>NAD(P)H + H^+ + quinone <=> $NAD(P)^+$ + hydroquinone	superoxide dismutase activity
SOD2	superoxide dismutase [Mn], mitochondrial	SOD2 catalyzes the dismutation of superoxide radical to hydrogen peroxide and oxygen in the mitochondrial matrix. SOD2 gene expression may be induced by FOXO activation, which occurs via deacetylation catalyzed by SIRT1.<br>Reaction:<br>2 $O_2^{\bullet -}$ + 2 H^+ <=> H_2O_2 + O_2	superoxide dismutase activity

ALDH7A1	alpha-aminoadipic semialdehyde dehydrogenase	Member of subfamily 7 in the aldehyde dehydrogenase family. It uses NAD^+ to catalyze the oxidation of aldehydes to the corresponding carboxylic acids. ALDH7A1 catalyzes the reaction in several metabolic pathways, such as fatty acid degradation, glycerolipid metabolism, amino acid metabolism. It metabolizes betaine aldehyde to the cellular osmolyte betaine. It is also active against lipid peroxidation-derived aldehydes. Located in cytoplasm.<br><br>Reaction:<br><br>aldehyde + NAD^+ + H_2O <=> carboxylic acid + NADH + H^+	cerebral organic acidurias, including diseases; amino acid metabolism
MDH2	malate dehydrogenase, mitochondrial	It catalyzes the reversible oxidation of malate to oxaloacetate in mitochondria, using NAD^+/NADH. It is part of the malate-aspartate shuttle, important for converting NADH generated in the cytosol into mitochondrial NADH.<br><br>Reaction:<br><br>Malate + NAD^+ <=> Oxaloacetate + NADH + H^+	cerebral organic acidurias, including diseases; urea cycle and associated pathways; NAD metabolism in oncogene-induced senescence and mitochondrial dysfunction-associated senescence; oxaloacetate metabolic process; amino acid metabolism; gluconeogenesis; glucose metabolism; metabolic reprogramming in colon cancer; glycolysis and gluconeogenesis
GOT1	aspartate aminotransferase, cytoplasmic	It is a pyridoxal phosphate-dependent enzyme with aminotransferase activity acting on several pathways: arginine biosynthesis; alanine, aspartate and glutamate metabolism; cysteine and methionine metabolism; arginine and proline metabolism; tyrosine metabolism; phenylalanine metabolism; phenylalanine, tyrosine and tryptophan biosynthesis. It is also part of the malate-aspartate shuttle.	urea cycle and associated pathways; NAD metabolism in oncogene-induced senescence and mitochondrial dysfunction-associated senescence; oxaloacetate metabolic process; amino acid metabolism; glutamate metabolic process; gluconeogenesis; glucose metabolism; glycolysis and gluconeogenesis

		Reactions:<br><br>1) L-aspartate + 2-oxoglutarate <=> oxaloacetate + L-glutamate<br>2) L-phenylalanine + 2-oxoglutarate <=> phenylpyruvate + L-glutamate<br>3) L-tyrosine + 2-oxoglutarate <=> 3-(4-hydroxyphenyl)pyruvate + L-glutamate<br>4) L-cysteine + 2-oxoglutarate <=> mercaptopyruvate + glutamate<br>5) L-cysteate + 2-oxoglutarate <=> 3-sulfopyruvate + L-glutamate<br>6) 3-sulfino-L-alanine + 2-oxoglutarate <=> 3-sulfinylpyruvate + L-glutamate<br>7) L-erythro-4-hydroxyglutamate + 2-oxoglutarate <=> (4R)-4-hydroxy-2-oxoglutarate + L-glutamate<br>8) (2S)-2-aminobutanoate + 2-oxoglutarate <=> 2-oxobutanoate + L-glutamate	
OAT	ornithine aminotransferase, mitochondrial	It is a pyridoxal phosphate-dependent enzyme with aminotransferase activity that catalyzes the reversible interconversion of L-ornithine and 2-oxoglutarate to L-glutamate semialdehyde and L-glutamate. It has a role in arginine and proline metabolism.<br><br>Reaction:<br><br>L-ornithine + 2-oxoglutarate <=> L-glutamate 5-semialdehyde + L-glutamate	urea cycle and associated pathways; glutamate and glutamine metabolism; amino acid metabolism; glutamate metabolic process; proline metabolic process; glutamine family amino acid biosynthetic process; proline biosynthetic process; L-proline biosynthetic process
G6PD	glucose-6-phosphate 1-dehydrogenase	It catalyzes the rate-limiting step of the oxidative pentose-phosphate pathway, which provides NADPH and pentose phosphates for fatty acid and nucleic acid synthesis.	NAD metabolism in oncogene-induced senescence and mitochondrial dysfunction-associated senescence; Cori cycle;

		Deacetylation by SIRT2 at Lys-403 stimulates its enzyme activity. Located in cytoplasm.<br><br>Reaction:<br><br>D-glucose 6-phosphate + $NADP^+$ => D-glucono-1,5-lactone 6-phosphate + NADPH + H^+	metabolic reprogramming in colon cancer; glycolysis in senescence
DUT	deoxyuridine 5'-triphosphate nucleotidohydrolase, mitochondrial	It catalyzes the cleavage of 2'-deoxyuridine 5'-triphosphate (dUTP) into 2'-deoxyuridine 5'-monophosphate (dUMP) and inorganic pyrophosphate, preventing uracil misincorporation into DNA and providing dUMP for de novo thymidylate biosynthesis. It also inhibits peroxisome proliferator-activated receptor (PPAR) activity.<br><br>Reaction:<br><br>dUTP + H_2O <=> dUMP + diphosphate	deoxyribose phosphate metabolic process; deoxyribose phosphate biosynthetic process; deoxyribonucleotide metabolic process; 2'-deoxyribonucleotide metabolic process; deoxyribonucleotide biosynthetic process; 2'-deoxyribonucleotide biosynthetic process
PNP	purine-nucleoside phosphorylase	It catalyzes the phosphorolysis of purine nucleosides, with the formation of the corresponding free purine base and pentose-1-phosphate. Involved in purine metabolism, nucleotide metabolism, and in nicotinate and nicotinamide metabolism. Nicotinamide riboside is converted to nicotinamide and ribose 1-phosphate by PNP. Located in cytoplasm.<br><br>Reactions:	deoxyribose phosphate metabolic process; deoxyribonucleotide metabolic process; 2'-deoxyribonucleotide metabolic process

		ribosylpurine + orthophosphate <=> purine + ribose 1-phosphate<br><br>purine deoxyribonucleoside + orthophosphate <=> purine + 2-deoxy-ribose 1-phosphate<br><br>nicotinamide riboside + orthophosphate <=> nicotinamide + ribose 1-phosphate	
RRM1	ribonucleoside-diphosphate reductase large subunit	It catalyzes the biosynthesis of deoxyribonucleotides from the corresponding ribonucleotides (ADP/GDP/CDP/UDP => dADP/dGDP/dCDP/dUDP). Deoxyribonucleotides are necessary for DNA replication and repair. Involved in purine, pyrimidine, glutathione, and nucleotide metabolism. Located in cytoplasm.<br><br>Reaction:<br><br>dADP + thioredoxin disulfide + H_2O <=> thioredoxin + ADP	deoxyribose phosphate metabolic process; deoxyribose phosphate biosynthetic process; deoxyribonucleotide metabolic process; 2'-deoxyribonucleotide metabolic process; deoxyribonucleotide biosynthetic process; 2'-deoxyribonucleotide biosynthetic process; ribonucleoside diphosphate metabolic process
ALDH18A1	delta-1-pyrroline-5-carboxylate synthase	Member of the aldehyde dehydrogenase family. It is a bifunctional ATP- and NADPH-dependent mitochondrial enzyme with both gamma-glutamyl kinase and gamma-glutamyl phosphate reductase activities. It is involved in the de novo biosynthesis of proline, ornithine, and arginine.<br><br>Reactions:	glutamate and glutamine metabolism; amino acid metabolism; glutamate metabolic process; proline metabolic process; glutamine family amino acid biosynthetic process; proline biosynthetic process; L-proline biosynthetic process

		gamma-glutamyl kinase activity: ATP + L-glutamate <=> ADP + L-glutamyl 5-phosphate<br><br>gamma-glutamyl phosphate reductase activity: L-glutamate 5-semialdehyde + orthophosphate + $NADP^+$ <=> L-glutamyl 5-phosphate + NADPH + H^+	
ADH5	alcohol dehydrogenase class-3	It catalyzes the oxidation of long-chain primary alcohols, long chain omega-hydroxy fatty acids (such as 20-HETE), and S-(hydroxymethyl) glutathione. Involved in fatty acid degradation, tyrosine metabolism, pyruvate metabolism. Located in cytoplasm.<br><br>Reactions:<br>1) a primary alcohol + NAD^+ <=> an aldehyde + H^+ + NADH<br>2) a secondary alcohol + NAD^+ <=> a ketone + H^+ + NADH<br>3) S-(hydroxymethyl)glutathione + $NAD(P)^+$ <=> S-formylglutathione + NAD(P)H + H^+<br>4) 20-oxo-(5Z,8Z,11Z,14Z)-eicosatetraenoate + H_2O + NAD^+ => (5Z,8Z,11Z,14Z)-eicosatetraenedioate + 2 H^+ + NADH<br>5) 20-hydroxy-(5Z,8Z,11Z,14Z)-eicosatetraenoate + NAD^+ = 20-oxo-(5Z,8Z,11Z,14Z)-eicosatetraenoate + H^+ + NADH	amino acid metabolism
PKM	pyruvate kinase PKM	It is a cytoplasmic enzyme that catalyzes the transfer of a phosphoryl group from phosphoenolpyruvate to ADP, generating ATP and pyruvate in the final rate-limiting step	amino acid metabolism; glycolysis; glucose metabolism; clear cell renal cell carcinoma pathways; metabolic reprogramming in colon cancer; aerobic glycolysis; glycolysis in senescence; glycolysis and gluconeogenesis; glycolytic process through

		of glycolysis. It can also phosphorylate pyruvate to phosphoenolpyruvate.<br>Reaction:<br>ATP + pyruvate <=> ADP + phosphoenolpyruvate	fructose-6-phosphate; canonical glycolysis; glycolytic process through glucose-6-phosphate; NADH regeneration; monosaccharide catabolic process; hexose catabolic process; nucleotide phosphorylation; glucose catabolic process; nucleoside diphosphate phosphorylation; purine nucleoside diphosphate metabolic process; ribonucleoside diphosphate metabolic process; glucose catabolic process to pyruvate; purine ribonucleoside diphosphate metabolic process; ATP generation from ADP; ADP metabolic process; glycolytic process
ALDOA	fructose-bisphosphate aldolase A	It catalyzes the reversible conversion of fructose 1,6-bisphosphate into two triose phosphate and plays roles in glycolysis, gluconeogenesis, pentose phosphate pathway, and fructose and mannose metabolism. Located in cytoplasm.<br>Reaction:<br>fructose 1,6-bisphosphate <=> glyceraldehyde 3-phosphate + dihydroxyacetone phosphate	glycolysis; gluconeogenesis; glucose metabolism; Cori cycle; clear cell renal cell carcinoma pathways; aerobic glycolysis; glycolysis and gluconeogenesis; fructose 1,6-bisphosphate metabolic process; fructose metabolic process; nucleotide phosphorylation; nucleoside diphosphate phosphorylation; purine nucleoside diphosphate metabolic process; ribonucleoside diphosphate metabolic process; purine ribonucleoside diphosphate metabolic process; ATP generation from ADP; ADP metabolic process; glycolytic process
ALDOC	fructose-bisphosphate aldolase C	It catalyzes the reversible aldol cleavage of fructose 1,6-bisphosphate and fructose 1-phosphate into dihydroxyacetone phosphate and either glyceraldehyde-3-phosphate or glyceraldehyde, respectively. It plays roles in glycolysis, gluconeogenesis, pentose phosphate pathway, and fructose and mannose metabolism. It is also involved in epithelial cell differentiation. Located in cytoplasm.	glycolysis; gluconeogenesis; glucose metabolism; clear cell renal cell carcinoma pathways; glycolysis in senescence; glycolysis and gluconeogenesis; fructose 1,6-bisphosphate metabolic process; fructose metabolic process; nucleotide phosphorylation; nucleoside diphosphate phosphorylation; purine nucleoside diphosphate metabolic process; ribonucleoside diphosphate metabolic process; purine

		Reactions:<br><br>fructose 1,6-bisphosphate <=> glyceraldehyde 3-phosphate + dihydroxyacetone phosphate<br><br>fructose 1-phosphate <=> glyceraldehyde + dihydroxyacetone phosphate	ribonucleoside diphosphate metabolic process; ATP generation from ADP; ADP metabolic process; glycolytic process
ENO1	alpha-enolase	It catalyzes the conversion of 2-phosphoglycerate to phosphoenolpyruvate in glycolysis. Also involved in the biosynthesis of amino acids. Located in cytoplasm.<br><br>Reaction:<br><br>2-phospho-D-glycerate <=> phosphoenolpyruvate + H_2O	glycolysis; gluconeogenesis; glucose metabolism; manipulation of host energy metabolism; clear cell renal cell carcinoma pathways; metabolic reprogramming in colon cancer; aerobic glycolysis; glycolysis in senescence; glycolysis and gluconeogenesis; glycolytic process through fructose-6-phosphate; canonical glycolysis; glycolytic process through glucose-6-phosphate; NADH regeneration; monosaccharide catabolic process; hexose catabolic process; nucleotide phosphorylation; glucose catabolic process; nucleoside diphosphate phosphorylation; purine nucleoside diphosphate metabolic process; ribonucleoside diphosphate metabolic process; glucose catabolic process to pyruvate; purine ribonucleoside diphosphate metabolic process; ATP generation from ADP; ADP metabolic process; glycolytic process
GPI	glucose-6-phosphate isomerase	In the cytoplasm it catalyzes the conversion of glucose-6-phosphate to fructose-6-phosphate, the second step in glycolysis, and the reverse reaction during gluconeogenesis. Extracellularly, it functions as a neurotrophic factor and as a lymphokine that induces immunoglobulin secretion. It is also a tumor-secreted cytokine and angiogenic factor.	glycolysis; gluconeogenesis; glucose metabolism; Cori cycle; clear cell renal cell carcinoma pathways; metabolic reprogramming in colon cancer; aerobic glycolysis; glycolysis and gluconeogenesis; nucleotide phosphorylation; nucleoside diphosphate phosphorylation; purine nucleoside diphosphate metabolic process; ribonucleoside diphosphate metabolic

		Reaction:<br><br>glucose 6-phosphate <=> fructose 6-phosphate	process; purine ribonucleoside diphosphate metabolic process; ATP generation from ADP; ADP metabolic process; glycolytic process
PGK1	phosphoglycerate kinase 1	It catalyzes the reversible conversion of 1,3-diphosphoglycerate to 3-phosphoglycerate in glycolysis, an ATP producing reaction. It may also act as a cofactor for polymerase alpha and is secreted by tumor cells where it participates in negative regulation of angiogenesis. It is also involved in epithelial cell differentiation. Located in cytoplasm.<br><br>Reaction:<br><br>ATP + 3-phosphoglycerate <=> ADP + 3-phospho-glyceroyl phosphate	glycolysis; gluconeogenesis; glucose metabolism; manipulation of host energy metabolism; Cori cycle; clear cell renal cell carcinoma pathways; metabolic reprogramming in colon cancer; aerobic glycolysis; glycolysis in senescence; glycolysis and gluconeogenesis; glycolytic process through fructose-6-phosphate; canonical glycolysis; glycolytic process through glucose-6-phosphate; NADH regeneration; monosaccharide catabolic process; hexose catabolic process; nucleotide phosphorylation; glucose catabolic process; nucleoside diphosphate phosphorylation; purine nucleoside diphosphate metabolic process; ribonucleoside diphosphate metabolic process; glucose catabolic process to pyruvate; purine ribonucleoside diphosphate metabolic process; ATP generation from ADP; ADP metabolic process; glycolytic process
FASN	fatty acid synthase	It catalyzes the biosynthesis of long-chain saturated fatty acids starting from acetyl-CoA and malonyl-CoA in the presence of NADPH. Located in cytoplasm. AMPK activation leads to decreased expression of FASN, repressing fatty acid synthesis.<br><br>Reaction:	clear cell renal cell carcinoma pathways; metabolic reprogramming in colon cancer

		acetyl-CoA + 2n H^+ + n malonyl-CoA + 2n NADPH => a long-chain fatty acid + n CO_2 + (n+1) CoA + 2n $NADP^+$	

[a]Source: Gene. National Center for Biotechnology Information. National Library of Medicine. National Institutes of Health. U.S. Department of Health and Human Services. https://www.ncbi.nlm.nih.gov/gene/ (accessed 2024-03-01 to 2024-04-20)

[b]Source: KEGG: Kyoto Encyclopedia of Genes and Genomes. https://www.genome.jp/kegg/ (accessed 2024-03-01 to 2024-04-20)

[c]The UniProt Consortium. UniProt: the universal protein knowledgebase. *Nucleic Acids Res*. **2018**, *46* (5), 2699. DOI: 10.1093/nar/gky092 (accessed 2024-03-01 to 2024-04-20)

Table S6. Twelve proteins associated to 36 representative terms and pathways selected among the significant terms and pathways of **Table S5** and **Figure S5**). Selection criteria: at least 2 genes from the loaded list associated with a term (Number Genes) and representing at least 5% of the total number of genes in the term (% Associated Genes). The proteins belong to the cluster of 35 less abundant proteins in the NR group.

Gene symbol	Protein name	Protein function[a,b,c]	Associated terms
HYOU1	hypoxia up-regulated protein 1	An endoplasmic reticulum protein involved in stress response. It belongs to the heat shock protein 70 family. Its suppression is associated with accelerated apoptosis. It has cytoprotective role in hypoxia-induced cellular perturbation. It is up regulated in tumors.	intrinsic apoptotic signaling pathway in response to hypoxia; negative regulation of endoplasmic reticulum stress-induced intrinsic apoptotic signaling pathway; response to ischemia
TP53	cellular tumor antigen p53	A tumor suppressor protein that responds to stress to regulate expression of target genes, inducing cell cycle arrest, apoptosis, senescence, DNA repair, or changes in metabolism. It induces the transcription of long intergenic non-coding RNA p21 (lincRNA-p21) and lincRNA-Mkln1. LincRNA-p21 participates in TP53-dependent transcriptional repression leading to apoptosis and seems to influence cell-cycle regulation.	intrinsic apoptotic signaling pathway in response to hypoxia; TP53 regulates transcription of additional cell cycle genes whose exact role in the p53 pathway remain uncertain; mammary gland development pathway - involution (Stage 4 of 4); hepatitis C and hepatocellular carcinoma; response to ischemia; negative regulation of post-transcriptional gene silencing; negative regulation of gene silencing by RNA; negative regulation of miRNA-mediated gene silencing; negative regulation of miRNA maturation; positive regulation of miRNA maturation; positive regulation of post-transcriptional gene silencing; positive regulation of post-transcriptional gene silencing by RNA; regulation of post-transcriptional gene silencing; regulation of gene silencing by RNA; negative regulation of post-transcriptional gene silencing by RNA; positive regulation of miRNA-mediated gene silencing; regulation of production of small RNA involved in gene silencing by RNA; regulation of post-transcriptional gene silencing by

			RNA; mRNA transcription; miRNA processing; regulation of miRNA-mediated gene silencing; regulation of miRNA maturation
HSPA1A or HSPA1B	heat shock 70 kDa protein 1A or 1B	Belong to the heat shock protein 70 family. Involved in stabilization of proteins against aggregation, and in folding of newly translated proteins. Involved in the ubiquitin-proteasome pathway. HSPA1A inhibition is proposed as a therapeutic approach for certain lymphomas.	negative regulation of endoplasmic reticulum stress-induced intrinsic apoptotic signaling pathway
P4HB	protein disulfide-isomerase	It catalyzes the formation, breakage, and rearrangement of -S-S- bonds in proteins. It acts as a chaperone that inhibits aggregation of misfolded proteins in a concentration-dependent manner. At low concentrations, facilitates aggregation of proteins (anti-chaperone activity). It is involved in hydroxylation of prolyl residues in preprocollagen.<br><br>It is up regulated in several cancer types[d].	interleukin-23 signaling; type I collagen synthesis in the context of osteogenesis imperfecta; protein hydroxylation; peptidyl-proline hydroxylation; peptidyl-proline hydroxylation to 4-hydroxy-L-proline; peptidyl-proline dioxygenase activity; procollagen-proline dioxygenase activity; peptidyl-proline 4-dioxygenase activity
STAT3	signal transducer and activator of transcription 3	It is activated through phosphorylation by the receptor associated kinases in response to various cytokines and growth factors. The activated protein acts as transcription activator in the cell nucleus. It mediates the expression of several genes, regulating cellular processes such as cell growth and apoptosis.<br><br>It is activated in different types of cancer and has a role in epithelial-mesenchymal transition (EMT)[e].	interleukin-23 signaling; mammary gland development pathway - involution (Stage 4 of 4); hepatitis C and hepatocellular carcinoma; negative regulation of post-transcriptional gene silencing; negative regulation of gene silencing by RNA; negative regulation of post-transcriptional gene silencing by RNA; negative regulation of miRNA-mediated gene silencing; negative regulation of miRNA maturation; positive regulation of post-transcriptional gene silencing; positive regulation of post-transcriptional gene silencing by RNA; regulation of post-transcriptional gene silencing; regulation of gene

			silencing by RNA; negative regulation of post-transcriptional gene silencing by RNA; positive regulation of miRNA-mediated gene silencing; regulation of production of small RNA involved in gene silencing by RNA; regulation of post-transcriptional gene silencing by RNA; mRNA transcription; miRNA processing; regulation of miRNA-mediated gene silencing; regulation of miRNA maturation
P4HA2	prolyl 4-hydroxylase subunit alpha-2	It catalyzes the post-translational formation of 4-hydroxyproline in -Xaa-Pro-Gly- sequences in collagens and other proteins, which is essential to the proper three-dimensional folding of procollagen chains.<br><br>It was verified that P4HA2 is highly expressed in lung adenocarcinoma tumor cells. High P4HA2 expression indicated a poor prognosis and served as an independent prognostic risk factor in lung cancer[f].<br><br>Reaction:<br><br>2-oxoglutarate + L-prolyl-[collagen] + O_2 <=> CO_2 + succinate + trans-4-hydroxy-L-prolyl-[collagen]	type I collagen synthesis in the context of osteogenesis imperfecta; protein hydroxylation; peptidyl-proline hydroxylation; peptidyl-proline hydroxylation to 4-hydroxy-L-proline; peptidyl-proline dioxygenase activity; procollagen-proline dioxygenase activity; peptidyl-proline 4-dioxygenase activity
SERPINH1	serpin H1<br><br>(HSP47)	A member of the serpin superfamily of serine proteinase inhibitors. It binds specifically to collagen and has a role as a chaperone in the biosynthetic pathway of collagen.<br><br>Its expression is associated with cancer growth and metastasis in several types of cancers. The presence of a high number of HSP47-positive fibroblasts in the cancer	type I collagen synthesis in the context of osteogenesis imperfecta

		stroma was a risk factor for recurrence of lung cancer after surgery[g].	
PSAT1	phosphoserine aminotransferase	It is a phosphoserine aminotransferase member of the class-V pyridoxal-phosphate-dependent aminotransferase family. Catalyzes the transamination of 3-phosphonooxypyruvate and L-glutamate to O-phosphoserine and 2-oxoglutarate in the pathway of L-serine biosynthesis.<br><br>It is overexpressed in tumors. Knockdown of *PSAT1* suppressed cell proliferation, migration, and invasion of non-small cell lung cancer (NSCLC)[h].<br><br>Reaction:<br><br>O-phospho-L-serine + 2-oxoglutarate <=> 3-phosphonooxypyruvate + L-glutamate	serine biosynthesis; serine metabolism; L-serine metabolic process; serine family amino acid biosynthetic process; L-serine biosynthetic process
PSPH	phosphoserine phosphatase	It belongs to a subfamily of the phosphotransferases. Catalyzes the last irreversible step in the biosynthesis of L-serine.<br><br>It is overexpressed in cancer, e.g. non-small cell lung cancer (NSCLC). Decreased expression of PSPH inhibited NSCLC cell migration, invasion, and proliferation[i].<br><br>Reaction:	serine biosynthesis; serine metabolism; L-serine metabolic process; serine family amino acid biosynthetic process; L-serine biosynthetic process

		O-phospho-L-serine + H_2O => L-serine + orthophosphate	
TNKS1BP1	182 kDa tankyrase-1-binding protein	Involved in cellular response to ionizing radiation; double-strand break repair; and positive regulation of protein phosphorylation.<br><br>It is overexpressed in cancer and correlated with poor overall survival in lung cancer patients. Its depletion inhibited cell epithelial-mesenchymal transition (EMT), proliferation, colony formation, migration, and invasion[j].	TP53 regulates transcription of additional cell cycle genes whose exact role in the p53 pathway remain uncertain
UCHL1	ubiquitin carboxyl-terminal hydrolase isozyme L1	It is a thiol protease that belongs to the ubiquitin C-terminal hydrolase family of deubiquitinases. It hydrolyzes a peptide bond at the C-terminal glycine of ubiquitin and eliminates ubiquitin isopeptide from target proteins. This process prevents protein degradation by the ubiquitin-proteasome system and is important for regulation of protein turnover in cells.<br><br>UCHL1 was shown to be up regulated in urothelial bladder carcinoma cells[k], and highly associated with the recurrence and invasion in breast cancer patients[l].	hepatitis C and hepatocellular carcinoma; response to ischemia
DDX5	probable ATP-dependent RNA helicase DDX5	Involved in the alteration of RNA structures, as a coregulator of transcription, a regulator of splicing, and in the processing of small noncoding RNAs. Contains the conserved Asp-Glu-Ala-Asp (DEAD) motif, important to ATP binding and hydrolysis as well as RNA binding and unwinding activities.	positive regulation of miRNA maturation; positive regulation of post-transcriptional gene silencing; positive regulation of post-transcriptional gene silencing by RNA; regulation of post-transcriptional gene silencing; regulation of gene silencing by RNA; positive regulation of miRNA-mediated gene silencing; regulation of production of small RNA involved in gene silencing by RNA;

		It has roles in global genome stability surveillance, DNA damage repair, and multiple oncogenic signaling pathways[m]. DDX5 overexpression is strongly correlated with aggressive tumor features of castration-resistant prostate cancer[n].	regulation of post-transcriptional gene silencing by RNA; mRNA transcription; miRNA processing; regulation of miRNA-mediated gene silencing; regulation of miRNA maturation

[a]Source: Gene. National Center for Biotechnology Information. National Library of Medicine. National Institutes of Health. U.S. Department of Health and Human Services. https://www.ncbi.nlm.nih.gov/gene/ (accessed 2024-03-01 to 2024-04-20)

[b]Source: KEGG: Kyoto Encyclopedia of Genes and Genomes. https://www.genome.jp/kegg/ (accessed 2024-03-01 to 2024-04-20)

[c]The UniProt Consortium. UniProt: the universal protein knowledgebase. *Nucleic Acids Res*. **2018**, *46* (5), 2699. DOI: 10.1093/nar/gky092 (accessed 2024-03-01 to 2024-04-20)

[d]Wang, F.; Yu, Z.; Liu, X.; Hu, F.; Liu, X.; Fu, X.; Liu, Y.; Zou, Z. A meta-analysis and bioinformatics analysis of P4HB expression levels in the prognosis of cancer patients. *Pathol. Res. Pract*. **2023**, *245*, 154474. DOI: 10.1016/j.prp.2023.154474

[e]Zhang, G.; Hou, S.; Li, S.; Wang, Y.; Cui, W. Role of STAT3 in cancer cell epithelial-mesenchymal transition (Review). *Int. J. Oncol*. **2024**, *64* (5), 48. DOI: 10.3892/ijo.2024.5636

[f]Lu, X. H.; Sang, D.; Zhang, Y. R.; Yuan, Q. High expression of prolyl 4-hydroxylase subunit alpha-2 in lung adenocarcinoma indicates poor prognosis. *Clinics* **2022**, *77*, 100123. DOI: 10.1016/j.clinsp.2022.100123

[g]Miyamura, T.; Sakamoto, N.; Ishida, K.; Kakugawa, T.; Taniguchi, H.; Akiyama, Y.; Okuno, D.; Hara, A.; Kido, T.; Ishimoto, H.; Miyazaki, T.; Matsumoto, K.; Tsuchiya, T.; Yamaguchi, H.; Miyazaki, T.; Obase, Y.; Ishimatsu, Y.; Nagayasu, T.; Mukae, H. Presence of heat shock protein 47-positive fibroblasts in cancer stroma is associated with increased risk of postoperative recurrence in patients with lung cancer. *Respir. Res*. **2020**, *21* (1), 234. DOI: 10.1186/s12931-020-01490-1

[h]Li, H.; Wu, C.; Chang, W.; Zhong, L.; Gao, W.; Zeng, M.; Wen, Z.; Mai, S.; Chen, Y. Overexpression of PSAT1 is correlated with poor prognosis and immune infiltration in non-small cell lung cancer. *Front. Biosci.* **2023**, *28* (10), 243. DOI: 10.31083/j.fbl2810243

[i]Liao, L.; Ge, M.; Zhan, Q.; Huang, R.; Ji, X.; Liang, X.; Zhou, X. PSPH mediates the metastasis and proliferation of non-small cell lung cancer through MAPK signaling pathways. *Int. J. Biol. Sci*. **2019**, *15* (1), 183-194. DOI: 10.7150/ijbs.29203

[j]Wang, S.; Guo, H.; Jia, J.; Zhang, W.; Gao, S.; Guan, H.; He, H.; Zhou, P. Silencing TAB182 inhibits cell EMT, migration and invasion by downregulating EGFR in A549 NSCLC cells. *Mol. Biol. Rep*. **2023**, *50* (4), 3073-3083. DOI: 10.1007/s11033-022-08176-5

[k]Zheng, Y.; Shi, D.; Chen, L.; Yang, Y.; Yao, M. UCHL1-PKM2 axis dysregulation is associated with promoted proliferation and invasiveness of urothelial bladder cancer cells. *Aging* **2023**, *15* (19), 10593-10606. DOI: 10.18632/aging.205097

[l]Mondal, M.; Conole, D.; Nautiyal, J.; Tate, E. W. UCHL1 as a novel target in breast cancer: emerging insights from cell and chemical biology. *Br. J. Cancer* **2022**, *126* (1), 24-33. DOI: 10.1038/s41416-021-01516-5

[m]Li, F.; Ling, X.; Chakraborty, S.; Fountzilas, C.; Wang, J.; Jamroze, A.; Liu, X.; Kalinski, P.; Tang, D. G. Role of the DEAD-box RNA helicase DDX5 (p68) in cancer DNA repair, immune suppression, cancer metabolic control, virus infection promotion, and human microbiome (microbiota) negative influence. *J. Exp. Clin. Cancer Res*. **2023**, *42* (1), 213. DOI: 10.1186/s13046-023-02787-x

[n]Le, T. K.; Cherif, C.; Omabe, K.; Paris, C.; Lannes, F.; Audebert, S.; Baudelet, E.; Hamimed, M.; Barbolosi, D.; Finetti, P.; Bastide, C.; Fazli, L.; Gleave, M.; Bertucci, F.; Taïeb, D.; Rocchi, P. DDX5 mRNA-targeting antisense oligonucleotide as a new promising therapeutic in combating castration-resistant prostate cancer. *Mol. Ther*. **2023**, *31* (2), 471-486. DOI: 10.1016/j.ymthe.2022.08.005

Table S7. Fragmentation and instrument parameters for the quantification of NAD^+, ATP, ADP, AMP, and lactate in BEAS-2B cells grown in monolayer by HPLC-ESI-MS/MS. DP = dissociation potential; CE = collision energy; CXP = collision cell exit potential.

Analyte	Q1	Q3	Dwell Time (msec)	DP (V)	CE (V)	CXP (V)
NAD 1	664.1	135.9	150	61	67	6
NAD 2	664.1	427.5	150	61	39	10
Benzamide	122.0	104.9	150	51	19	18
ATP 1	506.0	78.8	500	-65	-124	-17
ATP 2	506.0	159.0	500	-65	-48	-3
[$^{13}C_{10}{}^{15}N_5$]-ATP	521.0	78.8	500	-65	-124	-17
ADP 1	425.9	78.8	500	-70	-64	-13
ADP 2	425.9	158.7	500	-70	-42	-9
AMP 1	345.9	78.9	50	-50	-66	-1
AMP 2	345.9	96.8	50	-50	-20	-1
Lactate 1	88.9	45.0	50	-25	-14	-7
Lactate 2	88.9	43.0	50	-25	-18	-5
Benzoic acid	120.9	76.9	150	-45	-18	-1

Table S8. Precision of the analytical methods for quantification of NAD^+, ATP, ADP, AMP, and lactate in BEAS-2B cells by HPLC-ESI-MS/MS.

Analyte	Detected amount (pmol, mean ± SD)	Coefficient of variation (%)
NAD^+ (N = 7)	10.8 ± 0.7	6.5
ATP (N = 8)	84.0 ± 7.1	8.5
ADP (N = 8)	35.9 ± 5.4	15.1
AMP (N = 8)	2.9 ± 0.3	10.3
Lactate (N = 6)	488.4 ± 54.9	11.2

1a

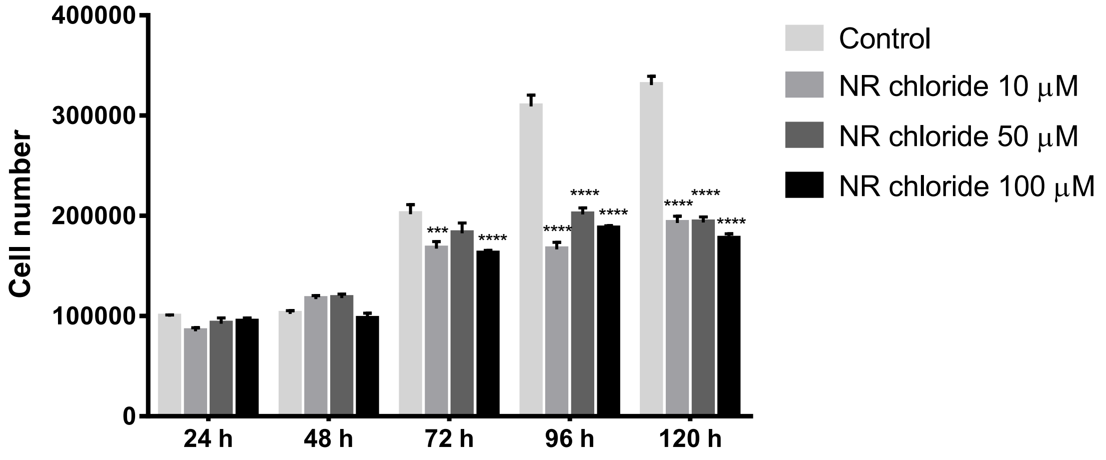

Cell number
400000
300000
200000
100000
0
24 h
48 h
72 h
96 h
120 h
***
****
Control
NR chloride 10 μM
NR chloride 50 μM
NR chloride 100 μM

1b

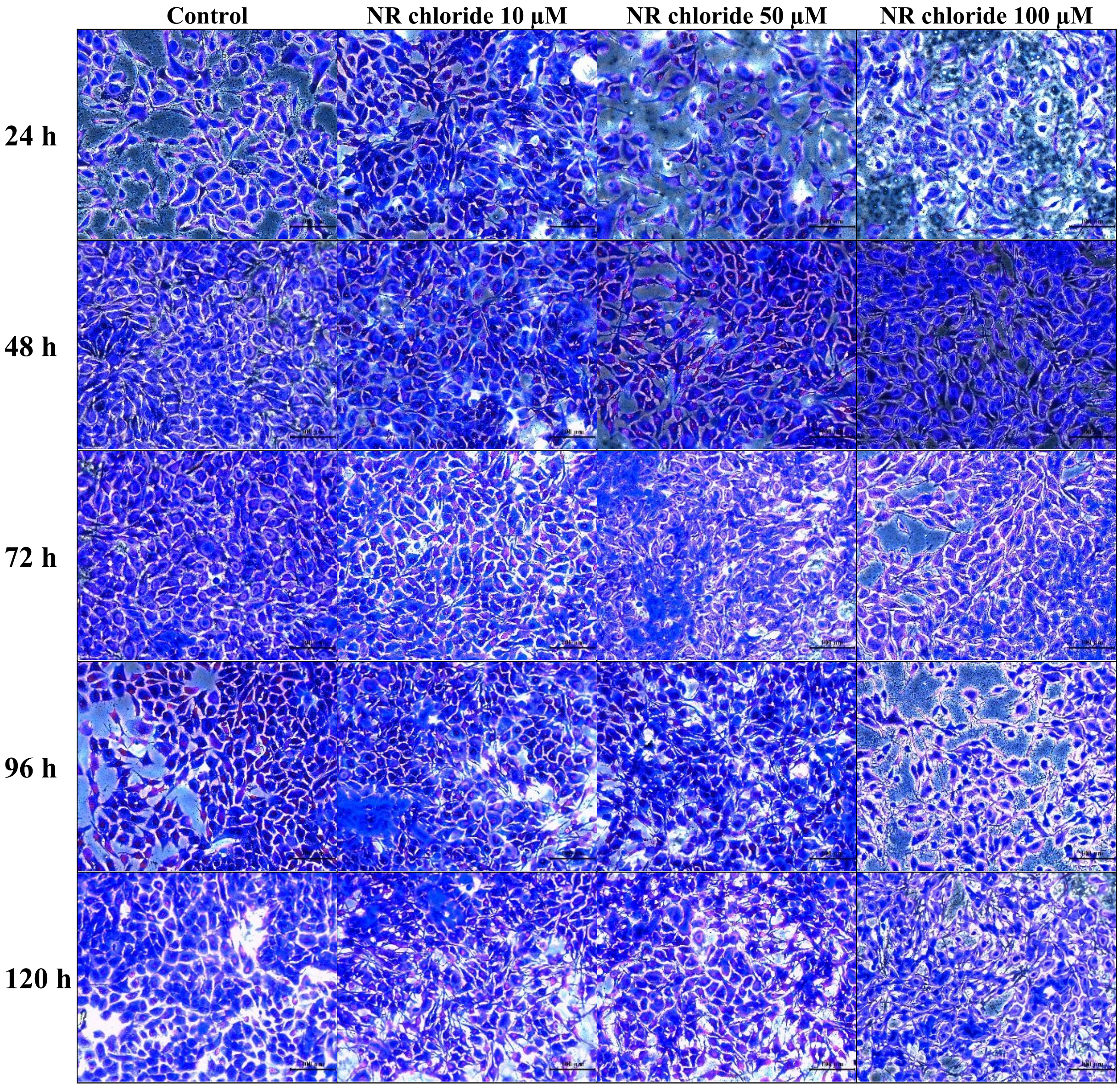

Control
NR chloride 10 μM
NR chloride 50 μM
NR chloride 100 μM
24 h
48 h
72 h
96 h
120 h

Figure S1. Nicotinamide riboside chloride (Sigma-Aldrich, Cat. number SMB00907, CAS number 23111-00-4) affects BEAS-2B cell growth. **a** Crystal violet dye (CVD) assay of the cells exposed to the indicated nicotinamide riboside chloride concentrations in the time range from 24 to 120 h. **b** Cell images obtained prior to the last step of the CVD assay at each time point (magnification: 200 x; scale bar: 100 µm). Panel a statistics: ordinary two-way ANOVA with Dunnett's multiple comparisons test, comparing the control group with the other groups within each time point, N = 7, ***$p < 0.001$, ****$p < 0.0001$. Plated cells: 4×10^4 cells/well.

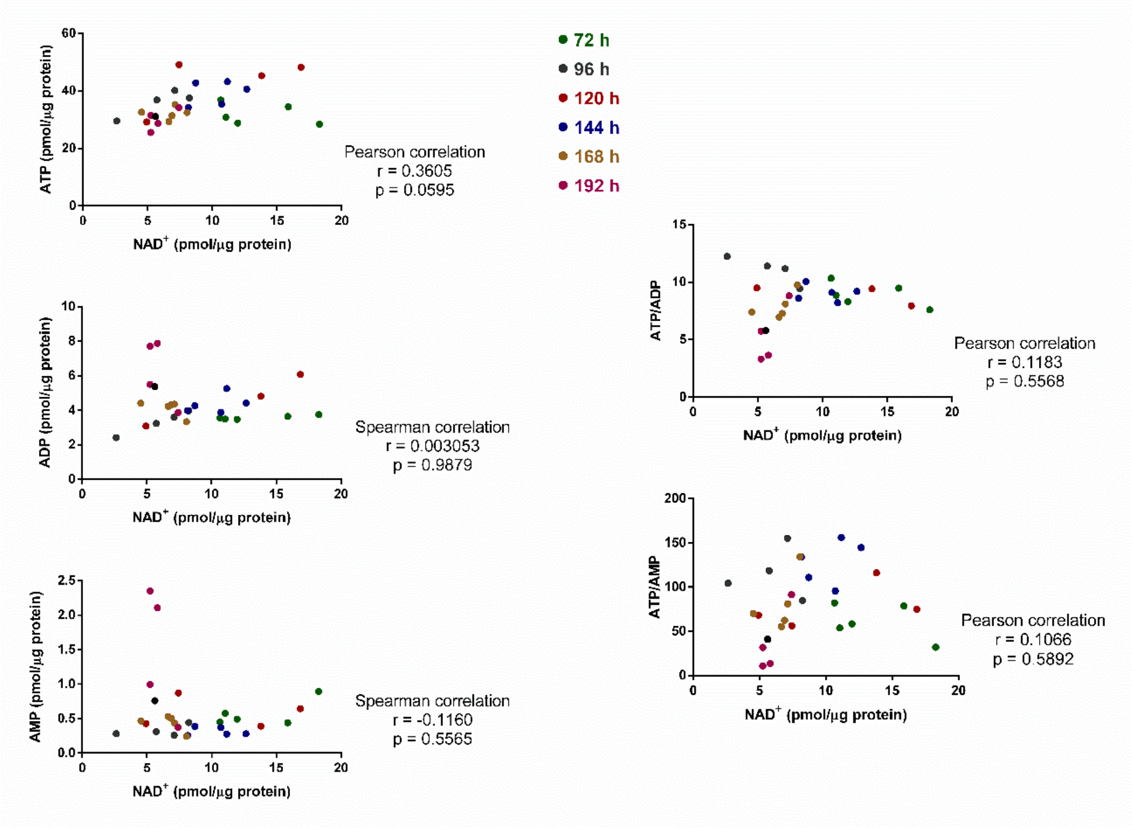

Figure S2. Correlation analyses of the levels of AMP, ADP, ATP, ATP/ADP and ATP/AMP with the NAD^+ content in the control cells in the 72 – 192 h interval.

3a

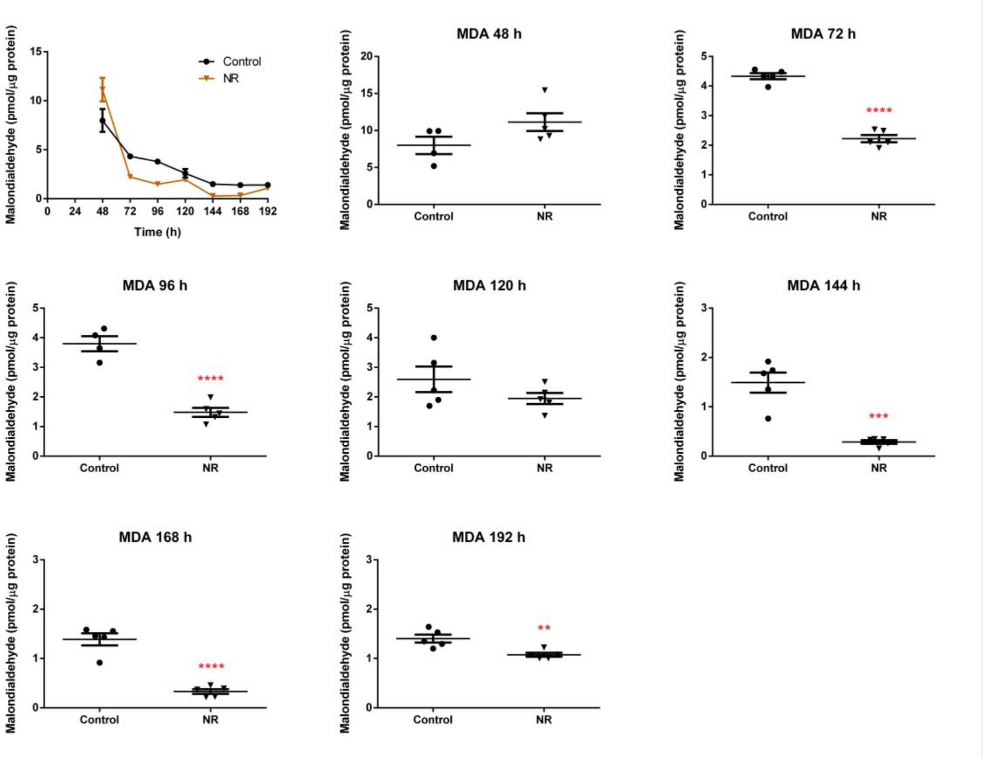

3b

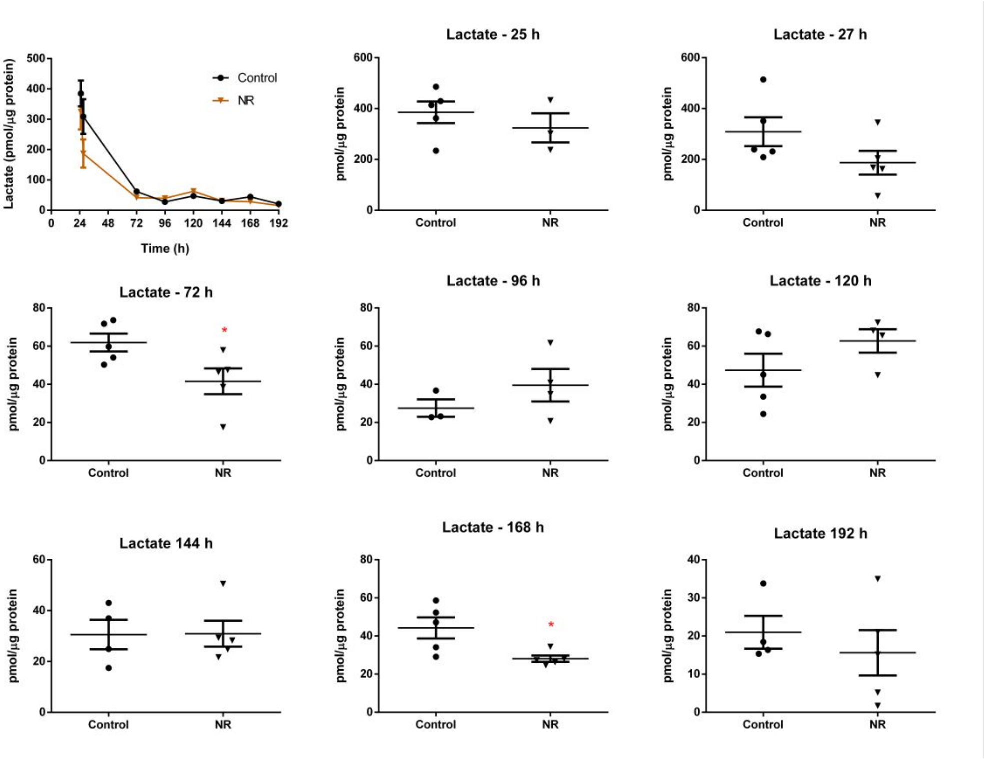

Figure S3. Nicotinamide riboside (NR) at the daily concentration of 1 µM protects BEAS-2B cells against redox stress (**a**) and does not increase lactate levels (**b**). The first panel in each figure gives the general view in the 24 – 192 h range. The other panels show

the comparisons between the control and the NR exposed cells in each time point. Unpaired t test, N = 3 to 5. *$p < 0.05$, ***$p < 0.001$, ****$p < 0.0001$. The experiment results are representative of two independent experiments.

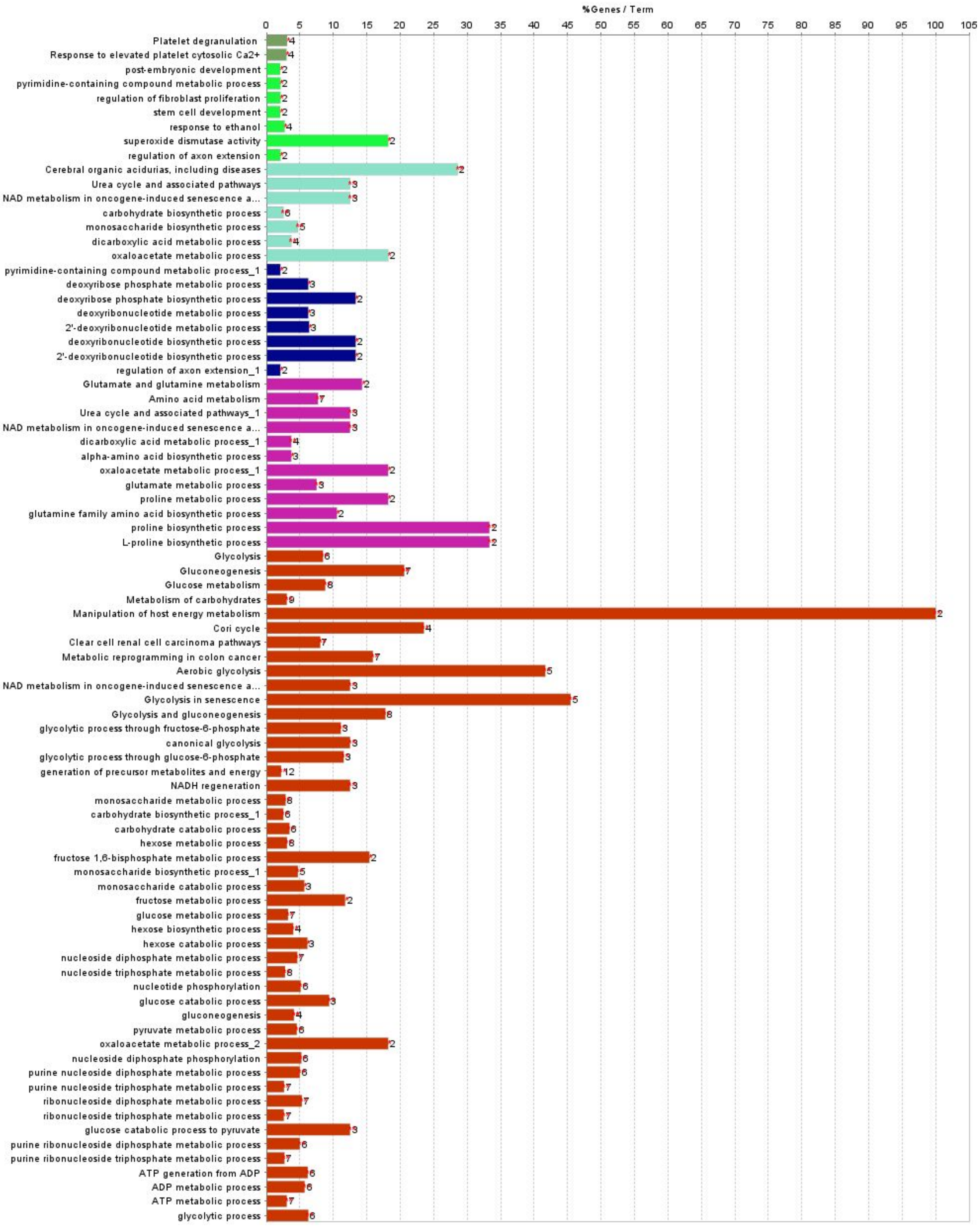

Figure S4. Nicotinamide riboside (NR) at the daily concentration of 1 μM modulates the abundance of proteins in BEAS-2B cells after 144 h of exposure in the culture grown in monolayer. Significant biological processes and pathways presenting at least 2 genes from the loaded list (42 more abundant proteins in the NR group) associated with a term (# genes in each bar) and representing at least 2% of the total number of genes in the term (% genes/term). The ClueGo tool (version 2.5.10) of the Cytoscape software (version

3.10.1) was used for pathway enrichment analyses. Ontologies selected: WikiPathways, REACTOME_Pathways, GO_BiologicalProcess. GO Fusion was not used to avoid losing information. The colors correspond to the groups shown in **Figure 9b**.

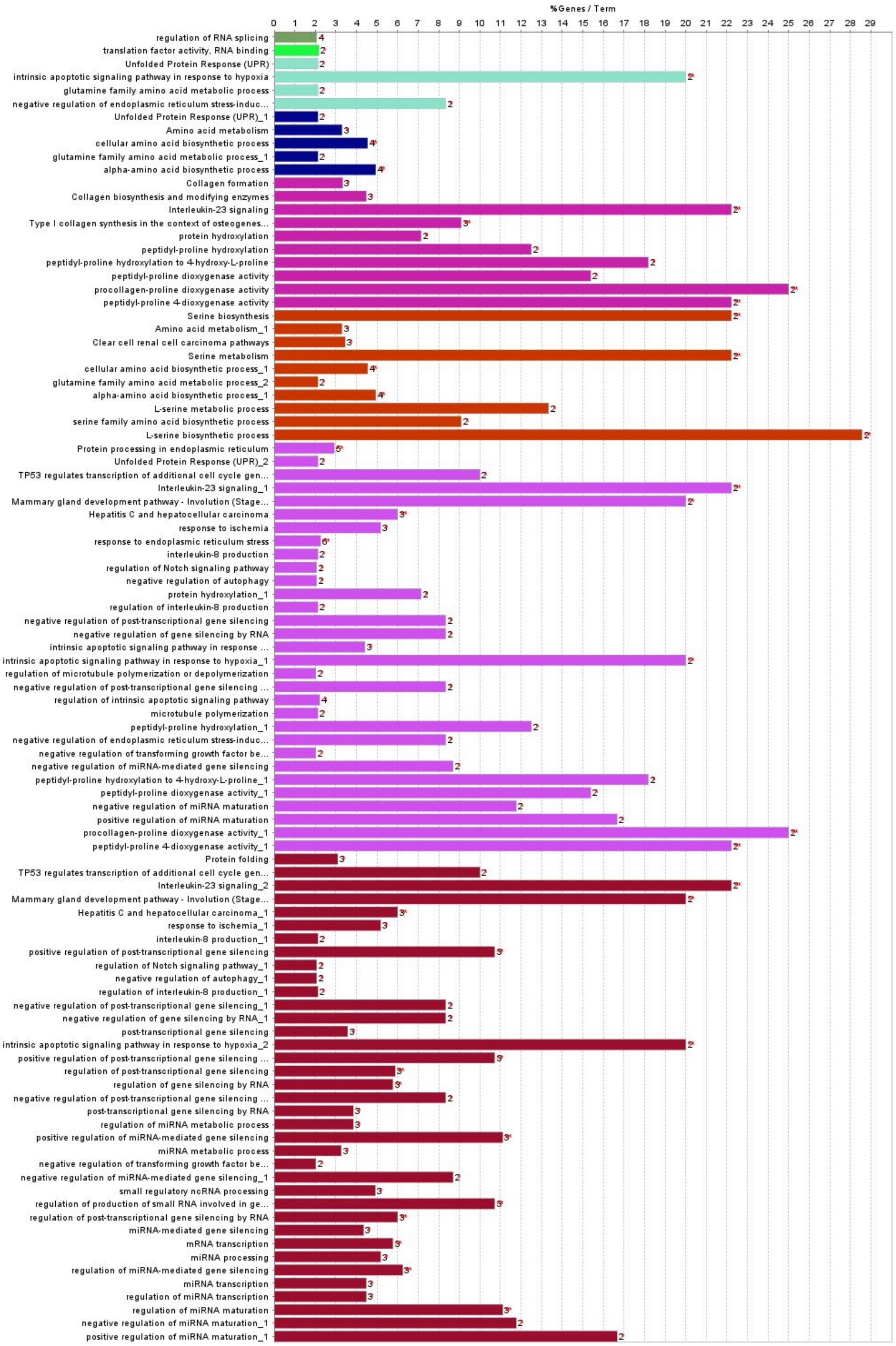
%Genes / Term
0 1 2 3 4 5 6 7 8 9 10 11 12 13 14 15 16 17 18 19 20 21 22 23 24 25 26 27 28 29
regulation of RNA splicing
translation factor activity, RNA binding
Unfolded Protein Response (UPR)
intrinsic apoptotic signaling pathway in response to hypoxia
glutamine family amino acid metabolic process
negative regulation of endoplasmic reticulum stress-induc...
Unfolded Protein Response (UPR)_1
Amino acid metabolism
cellular amino acid biosynthetic process
glutamine family amino acid metabolic process_1
alpha-amino acid biosynthetic process
Collagen formation
Collagen biosynthesis and modifying enzymes
Interleukin-23 signaling
Type I collagen synthesis in the context of osteogenes...
protein hydroxylation
peptidyl-proline hydroxylation
peptidyl-proline hydroxylation to 4-hydroxy-L-proline
peptidyl-proline dioxygenase activity
procollagen-proline dioxygenase activity
peptidyl-proline 4-dioxygenase activity
Serine biosynthesis
Amino acid metabolism_1
Clear cell renal cell carcinoma pathways
Serine metabolism
cellular amino acid biosynthetic process_1
glutamine family amino acid metabolic process_2
alpha-amino acid biosynthetic process_1
L-serine metabolic process
serine family amino acid biosynthetic process
L-serine biosynthetic process
Protein processing in endoplasmic reticulum
Unfolded Protein Response (UPR)_2
TP53 regulates transcription of additional cell cycle gen...
Interleukin-23 signaling_1
Mammary gland development pathway - Involution (Stage...
Hepatitis C and hepatocellular carcinoma
response to ischemia
response to endoplasmic reticulum stress
interleukin-8 production
regulation of Notch signaling pathway
negative regulation of autophagy
protein hydroxylation_1
regulation of interleukin-8 production
negative regulation of post-transcriptional gene silencing
negative regulation of gene silencing by RNA
intrinsic apoptotic signaling pathway in response ...
intrinsic apoptotic signaling pathway in response to hypoxia_1
regulation of microtubule polymerization or depolymerization
negative regulation of post-transcriptional gene silencing ...
regulation of intrinsic apoptotic signaling pathway
microtubule polymerization
peptidyl-proline hydroxylation_1
negative regulation of endoplasmic reticulum stress-induc...
negative regulation of transforming growth factor be...
negative regulation of miRNA-mediated gene silencing
peptidyl-proline hydroxylation to 4-hydroxy-L-proline_1
peptidyl-proline dioxygenase activity_1
negative regulation of miRNA maturation
positive regulation of miRNA maturation
procollagen-proline dioxygenase activity_1
peptidyl-proline 4-dioxygenase activity_1
Protein folding
TP53 regulates transcription of additional cell cycle gen...
Interleukin-23 signaling_2
Mammary gland development pathway - Involution (Stage...
Hepatitis C and hepatocellular carcinoma_1
response to ischemia_1
interleukin-8 production_1
positive regulation of post-transcriptional gene silencing
regulation of Notch signaling pathway_1
negative regulation of autophagy_1
regulation of interleukin-8 production_1
negative regulation of post-transcriptional gene silencing_1
negative regulation of gene silencing by RNA_1
post-transcriptional gene silencing
intrinsic apoptotic signaling pathway in response to hypoxia_2
positive regulation of post-transcriptional gene silencing ...
regulation of post-transcriptional gene silencing
regulation of gene silencing by RNA
negative regulation of post-transcriptional gene silencing ...
post-transcriptional gene silencing by RNA
regulation of miRNA metabolic process
positive regulation of miRNA-mediated gene silencing
miRNA metabolic process
negative regulation of transforming growth factor be...
negative regulation of miRNA-mediated gene silencing_1
small regulatory ncRNA processing
regulation of production of small RNA involved in ge...
regulation of post-transcriptional gene silencing by RNA
miRNA-mediated gene silencing
mRNA transcription
miRNA processing
regulation of miRNA-mediated gene silencing
miRNA transcription
regulation of miRNA transcription
regulation of miRNA maturation
negative regulation of miRNA maturation_1
positive regulation of miRNA maturation_1

Figure S5. Nicotinamide riboside (NR) at the daily concentration of 1 μM modulates the abundance of proteins in BEAS-2B cells after 144 h of exposure in the culture grown in monolayer. Significant biological processes and pathways presenting at least 2 genes from the loaded list (35 less abundant proteins in the NR group) associated with a term (# genes in each bar) and representing at least 2% of the total number of genes in the term (% genes/term). The ClueGo tool (version 2.5.10) of the Cytoscape software (version 3.10.1) was used for pathway enrichment analyses. Ontologies selected: WikiPathways, REACTOME_Pathways, GO_BiologicalProcess. GO Fusion was not used to avoid losing information. The colors correspond to the groups shown in **Figure 9c**.

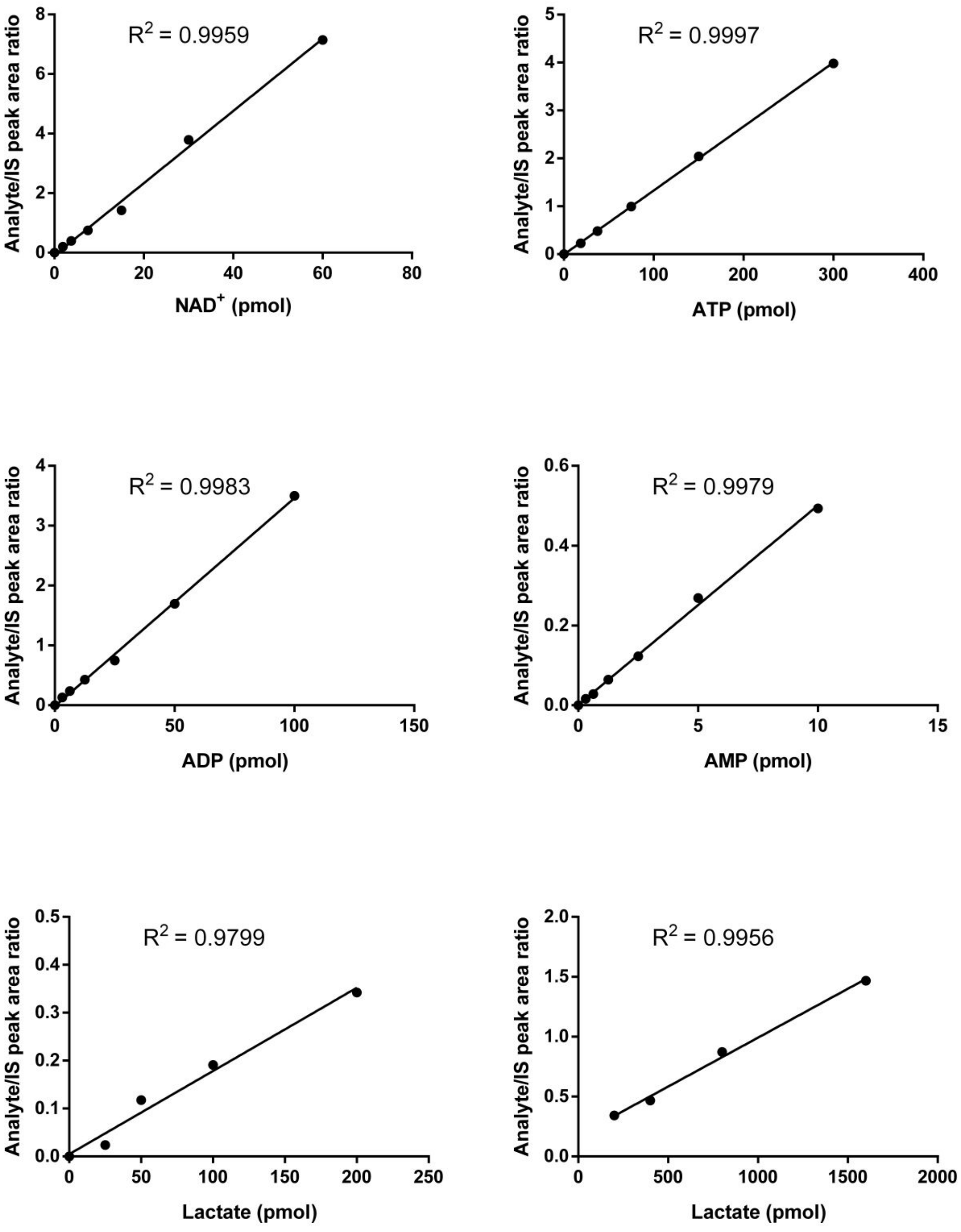

Figure S6. Calibration curves for quantification of NAD$^+$, ATP, ADP, AMP, and lactate in BEAS-2B cells by HPLC-ESI-MS/MS.
